# Design, Synthesis, and Evaluation of Alkyl-Quinoxalin-2(1*H*)-One Derivatives as Anti-*Quorum Sensing* Molecules, Inhibiting Biofilm Formation in *Aeromonas caviae* Sch3

**DOI:** 10.3390/molecules23123075

**Published:** 2018-11-24

**Authors:** René Blöcher, Ariel Rodarte Ramírez, Graciela Castro-Escarpulli, Everardo Curiel-Quesada, Alicia Reyes-Arellano

**Affiliations:** 1 Escuela Nacional de Ciencias Biológicas del Instituto Politécnico Nacional (ENCB-IPN), Departamento de Química Orgánica, Ciudad de México 11340, México; rene.bloecher@googlemail.com; 2 Escuela Nacional de Ciencias Biológicas del Instituto Politécnico Nacional (ENCB-IPN), Laboratorio de Investigación Clínica y Ambiental, Departamento de Microbiología, Ciudad de México 11340, México; rodar_t@hotmail.com (A.R.R.); chelacastro@hotmail.com (G.C.-E.); 3 Escuela Nacional de Ciencias Biológicas del Instituto Politécnico Nacional (ENCB-IPN), Departamento de Bioquímica, Ciudad de México 11340, México; ecqmixcoacdf@gmail.com

**Keywords:** *anti-quorum sensing*, biofilm, quinoxalin-2(1*H*)-one, *Aeromonas caviae*, bioisosteres, Grignard reaction

## Abstract

With the increasing antibiotic resistance of bacterial strains, alternative methods for infection control are in high demand. *Quorum sensing* (QS) is the bacterial communication system based on small molecules. QS is enables bacterial biofilm formation and pathogenic development. The interruption of QS has become a target for drug discovery, but remains in the early experimental phase. In this study, we synthesized a set of six compounds based on a scaffold (alkyl-quinoxalin-2(1*H*)-one), new in the anti-QS of Gram-negative bacteria *Aeromonas caviae* Sch3. By quantifying biofilm formation, we were able to monitor the effect of these compounds from concentrations of 1 to 100 µM. Significant reduction in biofilm formation was achieved by 3-hexylylquinoxalin-2(1*H*)-one (**11**), 3-hexylylquinoxalin-2(1*H*)-one-6-carboxylic acid (**12**), and 3-heptylylquinoxalin-2(1*H*)-one-6-carboxylic acid (**14**), ranging from 11% to 59% inhibition of the biofilm. This pilot study contributes to the development of anti-QS compounds to overcome the clinical challenge of resistant bacteria strains.

## 1. Introduction

The significant improvement of human health by the discovery of penicillin is undeniable. With the increase in bacterial strains resistant to virtually every clinically approved antibiotic, drug discovery research is facing the challenge of sustaining infection therapy [1,2]. The development of new antibiotics is limited and the result is often short lasting. Therefore, the need for alternative therapies has emerged and anti-quorum sensing represents a promising approach [1,2,3]. 

**Scheme 1:**
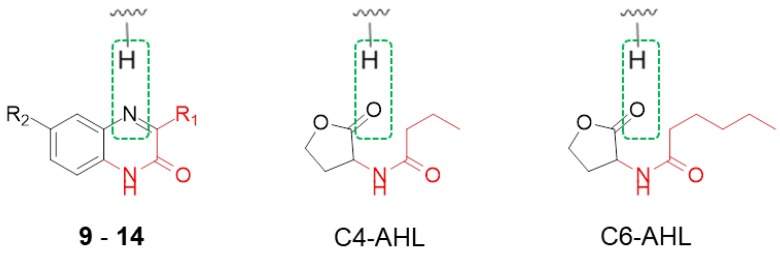
Design of *anti-quorum sensing* compounds based on the autoinducer structural characteristics, AHL, and bioisosteric replacement. The general core structure of the *anti-quorum sensing* compounds presented here is shown on the left. In the middle and on the right are illustrations of two *N*-acyl homoserine-lactone autoinducers (C4-AHL, C6-AHL) responsible for bacterial cell communication via *quorum sensing*. The *N*-acyl carbon chain is a consistent structural fragment in all three molecules and is marked in red. The bioisosteric proton acceptor substitution of the carbonyl moiety with the tertiary amine is highlighted with a green dashed line rectangle, visualizing the proton-binding area.

*Quorum sensing* describes the bacterial cell–cell communication based on small molecules called autoinducers [4]. Gram-negative bacteria regulate their virulence factors as a function of population cell density, which they sense by means of extracellular signal molecules such as *N*-acylated homoserine lactones (AHLs) in the QS system [5]. In Gram-negative bacteria, QS is also a key function for the formation of biofilm. Biofilm is a packed community of bacterial species embedded in a polymeric extracellular matrix providing channels for water and nutrients circulation [6]. The biofilm maintains bacterial viability, shields bacteria from antibiotic influences, and provides an environment for pathogen development [7]. *Aeromonas caviae* Sch3 is a Gram-negative bacterium and an environmental opportunistic pathogen of animals and humans [7]. *Aeromonas* cause gastroenteritis, wound infections, bacteremia, and, less frequently, respiratory infections, hepatobiliary infection, peritonitis, urinary tract infections, and ocular infections [7]. *Aeromonas* have a remarkable ability to colonize a broad variety of environments, relying on their biofilm production and cell–cell communication [7]. The disruption of biofilm formation is therefore a valuable target for novel anti-infective treatments. QS autoinducers are categorized into different types. For our investigation, only the type 1 autoinducer (AI-1) system, which is widespread among Gram-negative bacteria, is relevant [7]. The AI-1 system small molecules *N*-acyl homoserine lactones (AHLs), which are derived from common components of the bacterial metabolism [8]. The major autoinducers produced by *Aeromonas* are *N*-butanoyl homoserine lactone (C4-AHL) and *N*-hexanoyl homoserine lactone (C6-AHL) [9], shown in Scheme 1. To inhibit the QS mediated by these autoinducers, analogues containing increased *N*-acyl carbon chains (C10- to C14-AHL) have been used successfully [9]. There has been a focus on alterations of the *N*-acyl carbon chain within the QS inhibition of C4- and C6-AHL [10,11]. To develop innovative anti-QS compounds, we changed the lactone moiety of AHL by introducing a pharmacophores-oriented bioisoster [12,13], and to integrate the *N*-acyl carbon chain into a hetero cycle. Our structural solution resulted in the alkyl-quinoxalin-2(1*H*)-one shown in Scheme 1. We demonstrated its ability to significantly reduce biofilm formation in *Aeromonas caviae* Sch3 at the lowest compound concentration of 1 µM. 

## 2. Results and Discussion

### 2.1. Design of Quorum Sensing Inhibitors and Structural Development

The majority of *N*-acyl homoserine-lactone analogues provide their inhibition activity through an extended *N*-acyl carbon chain [14,15,16], while preserving the lactone moiety. As such, we decided to introduce a bioisosteric replacement of the lactones carbonyl proton acceptor with a tertiary amine as part of a closed ring heterocyclic structural solution. In Scheme 1, the pharmacophore and bioisosteres are marked with a green dashed-lined circle. By focusing on the pharmacophore proton-binding character of the autoinducer, changing the lactone fragment and introducing a bioisostere, we produced a valuable anti-quorum sensing scaffold. In our design, we adapted the *N*-acyl fragment of the *N*-acyl homoserine-lactone by integrating it into the 3-alkyl quinoxalin-2(1*H*)-one hetero cycle. In Scheme 1, the adapted structural motives are highlighted in red. This adaption enabled us to introduce different carbon chain lengths within our integrated *N*-acyl motive, inspired by the previous-stated importance of this structural element in the context of inhibitory strength [14,15,16].

During the structural development of these inhibitors, we realized a water solubility limitation under assay conditions with the hexyl carbon chain (**11**, 3-hexylylquinoxalin-2(1*H*)-one), so we were therefore not able to evaluate the heptyl carbon chain derivative (**13**, 3-hepylquinoxalin-2(1*H*)-one). To increase the water solubility and still evaluate the heptyl carbon chain derivative, we introduced a carboxylic acid functional group at position 6 (**14**, 3-heptylquinoxalin-2(1*H*)-one-6-carboxylic acid). The acid function was placed in a position where it should not interfere with the theoretical structure described above. To enable a direct comparison of acid and non-acid substitution in position 6, a derivative of the 3-hexylylquinoxalin-2(1*H*)-one (**11**), containing the acid substitution in position 6 (**12**, 3-hexylylquinoxalin-2(1*H*)-one-6-carboxylic acid), was synthesized and evaluated.

### 2.2. Synthesis

**Scheme 2:**
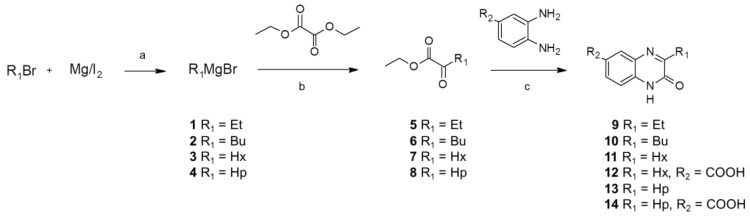
Synthesis pathway for 3-alkyl quinoxalin-2(1*H*)-one. (**a**) Dry THF, room temperature (rt; 25 °C) −40 °C, 45 min; (**b**) dry THF, –78 °C, 2 h; and (**c**) ethanol (EtOH), 12 h.

Scheme 2 shows the pathway for the synthesis of the anti-QS compounds (**9**–**14**). The synthetic route starts with the synthesis of required Grignard reagents (**1**–**4**) (Scheme 2a). Ethyl-, butyl-, hexyl-, and heptyl-bromide were reacted with magnesium turnings in tetrahydrofuran (THF) and a catalytic amount of iodine [17]. The activated Grignard reagents had to be used immediately at −78 °C with diethyl oxalate in dry THF (Scheme 2b). The resulting β-keto esters (**5**–**8**) were used without further purification in a condensation/cyclization reaction with *o*-diamino phenyl or 3-carboxyl *o*-diamino phenyl (Scheme 2c) [18]. The final 3-alkyl quinoxalin-2(1*H*)-one (**9**–**14**) were purified by column chromatography.

### 2.3. Inhibition of Biofilm Formation by Anti-Quorum Sensing Compounds 

**Figure 1:**
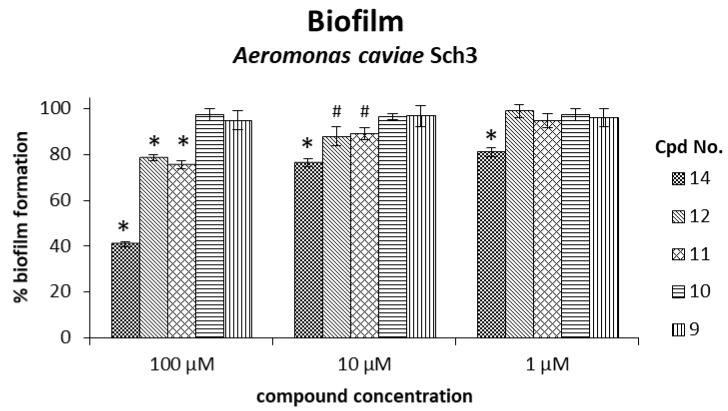
Biofilm formation influenced by compounds **9**, **10**, **11**, **12,** and **14** in *Aeromonas caviae* Sch3 at 6 × 10^8^ colony forming units (CFU)/mL bacteria. The data are presented as percentages of the biofilm formation of the control, which received no inhibitor treatment and was set as 100%. The compounds were evaluated at 100, 10, and 1 µM concentrations. Dimethyl sulfoxide (DMSO) had no significant effect on biofilm formation; the control experiment data can be found in the Supplementary Materials. Significance was confirmed by the Student’s *t*-test with an accuracy of * *p* < 0.005 and ^#^ *p* < 0.05 at the marked bars. Experiments were performed with *n* = 6. Compound number (Cpd No.)

The determination of the inhibition of biofilm formation in *A. caviae* Sch3 at a starting concentration of 6 × 10^8^ colony forming units (CFU)/ml bacteria, measured as absorbance of the culture at 570 nm, 0.50 absorbance (OD_570_ of 0.50) produced by five synthesized compounds (**9**–**12** and **14**, at 1, 10, and 100 µM) demonstrated a significant effect by compounds **11** and **12** at all but the lowest concentration and by **14** at all concentrations tested (Figure 1 and Table 1). The biofilm quantification was determined through measuring the absorbance at 570 nm of the dye staining the biofilm. Figure 1 shows the biofilm formation influenced by the five synthesized compounds. Table 1 shows the reduction in biofilm formation caused by the addition of the five synthesized compounds.

The most significant reduction in biofilm formation was achieved by 3-heptylquinoxalin-2(1*H*)-one-6-carboxylic acid (**14**), with a biofilm inhibition of 59% at 100 µM, 24% at 10 µM, and 19% at 1 µM (Table 1). 3-Hexylquinoxalin-2(1*H*)-one-6-carboxylic acid (**12**) and 3-hexylquinoxalin-2(1*H*)-one (**11**) showed similar and significant reduction in biofilm formation with 21% (**12**) and 25% (**11**) at a 100 µM compound concentration, as well as 12% (**12**) and 11% (**11**) inhibition at 10 µM. At 1 µM, both compounds (**11**, **12**) were not able to significantly decrease the biofilm formation, nor were 3-butylquinoxalin-2(1*H*)-one (**9**) or 3-ethylquinoxalin-2(1*H*)-one (**8**) able to significantly inhibit the biofilm formation at any used compound concentration.

From the analysis of the structure activity-relationship of our compound set, we concluded that the correlation of the *N*-acyl carbon chain length with biofilm inhibition reported in literature [14,15,16] and mentioned earlier in this work, is reflected in our results. The short length of our integrated *N*-acyl carbon chain, such as ethyl and butyl in compounds **9** and **10,** had no significant effect on biofilm formation, whereas compounds **11**, **12**, and **14** with extended hexyl and heptyl carbon chains showed significant inhibition. We additionally recognized that the introduction of a carboxylic acid functional group in position 6 increased water solubility, but did not affect biofilm inhibition in *Aeromonas caviae*. This was shown by the similar biofilm reduction of compounds **11** and **12**, whose structure only differs by the carboxylic acid substitution in position 6. This study provides anti-quorum sensing compounds for *Aeromonas caviae* Sch3 based on a scaffold benefiting future studies. 

### 2.4. Evaluation of Quorum Sensing Inhibition Through Reduction of Violacein Production in Chromobacterium Violaceum CV026 

**Figure 2:**
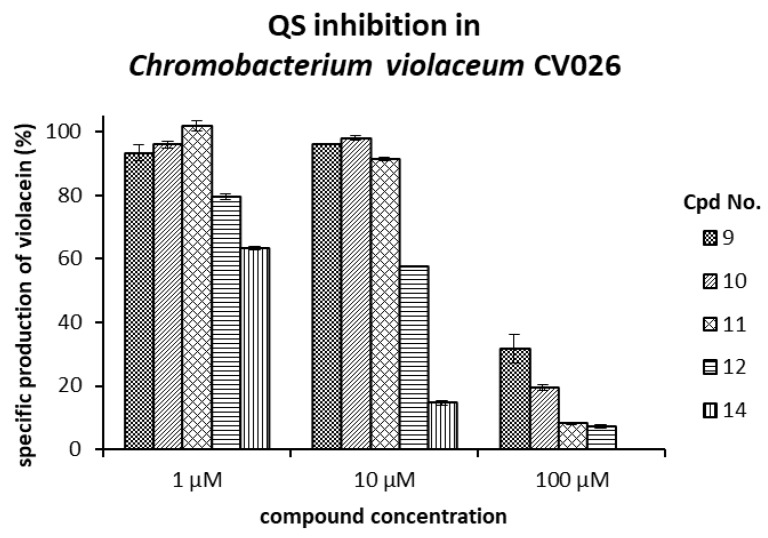
Production of violacein by *Chromobacterium violaceum* CV026 in the presence of compounds (**9**, **10**, **11**, **12**, and **14** at 1, 10, and 100 µM). The specific production of violacein was determined by the ratio of detectable pigment (OD_577_) per amount bacteria (OD_720_). The data were normalized on the untreated violacein production of the culture. Experiments were performed as duplicates. Compound number (Cpd No.).

The five synthesized compounds (**9**–**12** and **14**, at 1, 10, and 100 µM) were added to bacteria in L-broth, optical density at 600 nm, 0.1 absorbance (OD_600_ of 0.1) supplemented with C6-AHL and cells were incubated for 40 h. After quantifying violacein production from *Chromobacterium violaceum* CV026, we confirmed the QS inhibition observed before via the biofilm reduction in *Aeromonas caviae*. Figure 2 shows the specific production of violacein influenced by the added compounds.

The QS inhibition evaluated in *Chromobacterium violaceum* CV026 (Figure 2) describes a similar trend as the QS inhibition in *Aeromonas caviae* (Figure 1), which was demonstrated via biofilm reduction. The violacein production in *Chromobacterium violaceum* CV026 is a more sensitive assay for QS inhibition than the quantification of biofilm formation. Therefore, stronger QS inhibition is reported in Figure 2. Compound **14** shows the most significant effect throughout all tested concentrations. The major effects of all five compounds were observed at the highest concentration of 100 µM, with a clearly recognizable dose-dependent response. 

## 3. Materials and Methods 

### 3.1. Chemistry

All reagents and solvents were purchased from Sigma Aldrich, Toluca, Mexico and used directly without further purification. All reactions were carried out at room temperature, if not stated otherwise. Reactions were monitored by thin-layer chromatography (TLC) on Merck F_253_ silica gel aluminum sheets, and spots were revealed with ultraviolet (UV) light (254 nm) (Darmstadt, Mercury apparatus at 300 MHz and 75 MHz, respectively, or on a Varian at 500 and 125 MHz, respectively (Palo Alto, CA, USA). The chemical shifts (*δ*) are referenced to internal (CH_3_)_4_Si with *δ* ^1^H = 0, *δ* ^13^C = 0, and given in ppm. High Resolution Mass Spectra (HRMS) were recorded on a Bruker micrOTOF Q and processed with the Software Bruker Compass DataAnalysis 4.1 (Karlsruhe, Germany).

#### 3.1.1. General Method for the Synthesis of Compounds **9**–**14**, Described at the Example of 3-Ethylquinoxalin-2(1*H*)-one (**8**)

Magnesium turnings (Mg°), 0.2 g (8 mmol), were washed with 100 mL hexane by repeated decanting. The Mg° was dried in vacuum under heat support. The Mg° was allowed to cool down while still under vacuum. The reaction vessel, a 25-mL 2-neck round bottom flask, was supplied with a dropping-funnel and sealed with septa. The apparatus was flushed with nitrogen gas while heated with a heat-gun. The apparatus was allowed to cool down while still flushed in a nitrogen stream. The dried Mg° was transferred into the dry apparatus. A catalytic amount of iodine was added to the metal as a reaction indicator [17] and catalyst [19]. In the dropping-funnel above, we placed 0.6 mL (8 mmol) 1-bromoethan in 8 mL THF (1 molar concentration). Under stirring in the nitrogen atmosphere, 1–2 mL of the halide solution were added to the magnesium-iodine, just enough to cover it. To start the reaction, the magnesium was cracked with a spatula or glass rod to break the magnesium-oxide layer while covered with THF. Silver shining magnesium became visible. Under strong stirring, the reaction started within 15 min (long chain alkyl bromides needed up to a maximum of 45 min before reaction onset). Activation of the reagent was observed by a color change of the iodine. From an initial orange, the mixture turned into yellow and the color finally disappeared, leaving a grey solution. At this point, the remaining alkyl-halide solution was added slowly dropwise to the round-bottom flask [17]. After completing addition, the mixture was heated to 40 °C and stirred for another 30 min to guarantee total activation. The magnesium turnings disappeared and a cloudy grey solution remained with few black particles. For the next step, a second apparatus was prepared. One 100-mL round-bottom flask with a dropping-funnel on top was placed in a Dewar and dried as previously described in a nitrogen stream. In the round-bottom flask, we placed 0.98 mL (7.2 mmol) diethyl oxalate in 3 mL dry THF and cooled down to −78 °C in a dry-ice/acetone bath. After the Grignard reagent was activated, it was transferred into the second dropping-funnel. Here, it was important to work quickly and precisely as the reagent becomes inactivated by air and moisture. Likewise, side products can be produced, such as salts that can block the second dropping funnel, if addition is postponed too long. After the active Grignard reagent was placed in the second dropping-funnel, a slow/drop-wise addition into the −78 °C cold round-bottom flask followed. A fast addition of the Grignard reagent resulted in the occurrence of side products. After the addition was completed, the mixture was stirred at −78 °C for 2 h, followed by a fast quenching of the reaction at −78 °C with 20 mL of 3 N hydrochloric acid aqueous solution. The reaction was allowed to warm up to room temperature, and was subsequently extracted with 50 mL of dichloromethane. The organic layer was washed with water, dried over sodium sulfate, and removed under reduced pressure, leaving a clear liquid—the ketovaline ethyl ester (**1**)—which was used without further purification. Then, 0.78 g (7.2 mmol) *O*-phenylendiamine were dissolved in 6 mL ethanol under nitrogen atmosphere [18]. The synthesized ketovaline ethyl ester (**1**) was dissolved in 2 mL ethanol and added slowly to the *o*-phenylendiamine ethanolic solution. The reaction was stirred for 12 h. The 3-ethylquinoxalin-2(1*H*)-one (**9**) precipitated in the reaction solution. The crude product was collected through filtration and washed with 3–5 mL cold ethanol. Final purification was performed by column chromatography with silica gel and a solvent mixture of ethyl acetate/hexane 1/2. In the purification process of the acidic derivatives, 3-hexylquinoxalin-2(1*H*)-one-6-carboxylic acid (**12**), and 3-heptylquinoxalin-2(1*H*)-one-6-carboxylic acid (**14**), 1% acetic acid was added to the chromatography eluent mixture. The pure compounds all remained as white solid powder. 

#### 3.1.2. Analytics

##### 3-Ethylquinoxalin-2(1*H*)-one (**9**)


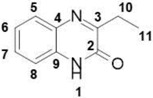


Total yield: 29%. ^1^H NMR (500 MHz, dmso) *δ* 12.31 (s, 1H; **1**), 7.71 (dd, *J* = 7.9, 0.7 Hz, 1H; **8**), 7.49–7.43 (m, 1H; **5**), 7.29–7.23 (m, 2H; **6**, **7**), 2.79 (q, *J* = 7.4 Hz, 2H; **10**), 1.23–1.19 (m, 3H; **11**). ^13^C NMR (126 MHz, DMSO) δ 167.76 (**2**), 159.77 (**3**), 139.98 (**9**), 136.86 (**4**), 134.63 (**5**), 128.23 (**6**), 122.58 (**7**), 119.82 (**8**), 27.9 (**10**), 9.5 (**11**); IR: KBr *v*: 3436 (N–H) cm^−1^, 2946 (C–H) cm^−1^, 1663 (C=O) cm^−1^, 1567 (N=C) cm^−1^; HRMS: measured 174.0873 (calculated for C_10_H_10_N_2_O: 174.0866 ).

##### 3-Butylquinoxalin-2(1*H*)-one (**10**)


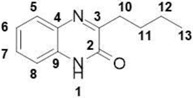


Total yield: 55%. ^1^H NMR (500 MHz, DMSO) *δ* 12.34 (s, 1H; **1**), 7.73 (d, *J* = 7.8 Hz, 1H; **8**), 7.48 (d, *J* = 7.7 Hz, ^1^H; **5**), 7.28 (t, *J* = 8.1 Hz, 2H; **6**, **7**), 2.83–2.75 (m, 2H; **10**), 1.74–1.64 (m, 2H; **11**), 1.47–1.34 (m, 2H; **12**), 0.98–0.89 (m, 3H; **13**). ^13^C NMR δ 161.88 (**2**), 154.58 (**3**), 131.74 (**9**), 129.36 (**4**), 128.06 (**8**), 123.06 (**7**), 112.91 (**5**), 115.21 (**6**), 28.26 (**10**), 22.10 (**11**), 18.59 (**12**), 13.90 (**13**); IR: KBr *v*: 3436 (N–H) cm^−1^, 2946 (C–H) cm^−1^, 1663 (C=O) cm^−1^, 1562 (N=C) cm^−1^; HRMS: measured 203.1118 (calculated for C_12_H_14_N_2_O: 203.1179).

##### 3-Hexylquinoxalin-2(1*H*)-one (**11**)


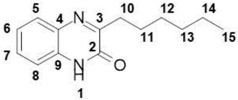


Total yield: 42%. ^1^H NMR (500 MHz, DMSO) *δ* 12.30 (s, 1H; **1**), 7.74–7.66 (m, 1H; **8**), 7.52–7.41 (m, 1H; **5**), 7.31–7.22 (m, 2H; **6**, **7**), 2.82–2.69 (m, 2H; **10**), 1.65 (qd, *J* = 14.9, 7.5 Hz, 2H; **11**), 1.44–1.13 (m, 6H; **12**, **13**, **14**), 0.91–0.79 (m, 3H; **15**). ^13^C NMR (126 MHz, DMSO) *δ* 167.26 (**2**), 160.01 (**3**), 136.90 (**4**), 136.82 (**9**), 134.47 (**5**), 133.37 (**6**), 128.23 (**7**), 120.41 (**8**), 33.66 (**12**), 33.76 (**13**), 31.21 (**10**), 27.22 (**11**), 22.9 (**14**), 19.15 (**15**); IR: KBr *v*: 2951 (C–H) cm^−1^, 1667 (C=O) cm^−1^, 1562 (N=C) cm^−1^; HRMS: measured 231.1496 (calculated for C_14_H_18_N_2_O: 231.1492 ).

##### 3-Hexylquinoxalin-2(1*H*)-one-6-carboxylic acid (**12**)


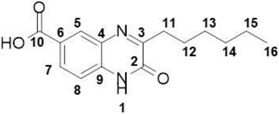


Total yield: 21%. ^1^H NMR (500 MHz, DMSO) *δ* 13.24 (s, 1H; **10**), 12.52 (s, 1H; **1**), ), 7.97 (dd, *J* = 8.5, 1.9 Hz, 1H; **5**), 7.87–7.72 (m, 1H; **7**), 7.35–7.27 (m, 1H; **8**), 2.81–2.69 (m, 2H; **11**), 1.71–1.60 (m, 2H; **12**), 1.38–1.15 (m, 6H; **13**, **14**, **15**), 0.89–0.77 (m, 3H; **16**). ^13^C NMR (126 MHz, DMSO) δ 167.07, 163.36, 155.12, 135.56, 131.32, 130.23, 129.88, 125.83, 117.09, 115.83, 31.61, 29.17, 28.94, 22.46, 14.35; IR: KBr *v*: 3430 (N–H) cm^−1^, 1658 (C=O) cm^−1^, HRMS: measured 274.3169 (calculated for C_15_H_18_N_2_O_3_: 274.3150).

##### 3-Heptylquinoxalin-2(1*H*)-one (**13**)


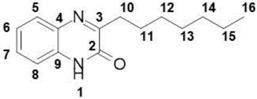


Total yield: 27%. ^1^H NMR (500 MHz, DMSO) *δ* 12.30 (s, 1H; **1**), 7.70 (d, *J* = 7.5 Hz, 1H; **8**), 7.49–7.43 (m, 1H; **5**), 7.25 (dd, *J* = 12.0, 4.5 Hz, 2H; **6**, **7**), 2.80–2.69 (m, 2H; **10**), 1.68 (dt, *J* = 15.2, 7.5 Hz, 2H; **11**), 1.44–1.14 (m, 8H; **12**, **13**, **14**, **15**), 0.83 (dt, *J* = 14.8, 8.8 Hz, 3H; **16**). ^13^C NMR (126 MHz, DMSO) δ 167.06 (**2**), 159.77 (**3**), 136.90 (**9**), 136.82 (**4**), 134.46 (**6**), 133.23 (**5**), 128.28 (**7**), 120.40 (**8**), 36.43 (**14**), 34.03 (**13**), 33.67 (**12**), 31.25 (**10**), 27.28 (**11**), 23.8 (**15**), 19.11 (**16**); IR: KBr *v*: 3436 (N–H) cm^−1^, 2915 (C–H) cm^−1^, 1663 (C=O) cm−1, 1565 (N=C) cm^−1^; HRMS: measured 245.1650 (calculated for C_15_H_20_N_2_O: 245.1648).

##### 3-Heptylquinoxalin-2(1*H*)-one-6-carboxylic acid (**14**)


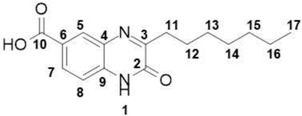


Total yield: 23%. ^1^H NMR (500 MHz, DMSO) *δ* 13.09 (s, 1H; **10**), 12.63 (s, 1H; **1**), 8.12–8.04 (m, 1H; **5**), 7.99–7.74 (m, 1H; **7**), 7.51–7.32 (m, 1H; **8**), 2.90–2.82 (m, 2H; **11**), 1.82–1.73 (m, 2H; **12**), 1.48–1.27 (m, 8H; **13**, **14**, **15**, **16**), 0.98–0.88 (m, 3H; **17**); ^13^C NMR (126 MHz, DMSO) δ 166.06 (**10**), 163.9 (**2**), 157.67 (**3**), 137.91 (**9**), 135.92 (**4**), 134.25 (**6**), 131.23 (**5**), 129.28 (**7**), 123.40 (**8**), 35.43 (**14**), 33.23 (**13**), 31.67 (**15**), 30.23 (**11**), 26.18 (**12**), 21.5 (**16**), 17.09 (**17**); IR: KBr *v*: 3414 (N–H) cm^−1^, 2915 (C–H) cm^−1^, 1661 (C=O) cm−1, 1568 (N=C) cm^−1^; HRMS: measured 289.1549 (calculated for C_16_H_20_N_2_O_3_: 289.1547). Selected Spectra: IR, ^1^H, ^13^C, HSQC, HMBC and HRMS can be found in the Supplementary Materials.

### 3.2. Bacteriology

For the evaluation of biofilm reduction in *Aeromonas caviae* Sch3, previous published protocols were used [20]. A brief description of the process is provided here as well as individual changes. 

#### 3.2.1. Bacterial Strain *Aeromonas caviae* Sch3

We used the *Aeromonas caviae* Sch3 strain isolated from diarrheal feces of a 5-year-old child patient, kindly provided by Dr. Jonathan Shaw from the University of Sheffield Medical School (Sheffied, United Kingdom). Genetic and biochemical characterization of *A. caviae* Sch3 was performed by Dr. Castro-Escarpulli from the “Escuela Nacional de Ciencias Biólogicas del Instituto Politécnico Nacional” (Mexico City, Mexico).

#### 3.2.2. Culture Conditions *Aeromonas caviae* Sch3

*Aeromonas caviae* Sch3 strain was grown on 1.5% trypticase soy agar medium (TSA) (Bioxon, Mexico City, Mexico) at 37 °C for 16 h. Short-term storage of isolates was carried out in minimal maintenance medium: 1% *v*/*v* polypeptone, 0.3% *v*/*v* yeast extract, 0.5% *v*/*v* bacteriological agar, and 0.85% *v*/*v* NaCl) at room temperature (RT). Long-term storage was carried out in Todd Hewitt broth (Oxoid, Mexico City, Mexico) containing 30% (*v*/*v*) glycerol at –70 °C.

#### 3.2.3. Evaluation of Biofilm Reduction

In transparent flat bottom 24-well plates, *Aeromonas caviae*-Sch3 was used at a concentration of 6 × 10^8^ CFU/mL. Bacterial concentration was adjusted to an OD_570_ of 0.50. Compounds **9**, **10**, **11**, **12**, and **14** were evaluated at 100, 10, and 1 µM, and compared to a control of the exact same mixture without compound within the same plate. Each well contained a volume of 2000 µL. For the compound evaluation, the composition consisted of 1880 µL broth (with 1% TWEEN80), 20 µL compound DMSO stock of 10, 1, and 0.1 mM concentration (resulting in assay concentrations of 100, 10, and 1 µM with 1% DMSO, respectively), and 100 µL of 6 × 10^8^ CFU/mL bacteria in broth. Plates were incubated for 16 h at 37 °C and processed as previously described [20]. Briefly, all the liquid was disposed. The remaining biofilm attached to the surface was washed once with distilled water and fixed with absolute methanol. The dried biofilm was stained with 2.5 mL per well of 0.4% (*w*/*v*) crystal violet solution for 15 min at room temperature. After eliminating excess staining solution from the wells, the attached dye was dissolved from the biofilm with 2.5 mL 33% glacial acetic acid solution. The absorbance of the obtained solution, equivalent to the biofilm quantity, was detected in an Epoch BioTek (Winooski, VT, USA) reader at 570 nm and visualized with the Gen5 software (Bio Tek, Winooski, VT, USA). Experiments were performed with *n* = 6, and statistical significance was evaluated with the Student’s *t*-test in Microsoft Office (Redmond, WA, USA).

#### 3.2.4. Culture Conditions *Chromobacterium violaceum* CV026

Luria Bertani (LB) broth was prepared in 1 L of distilled water by adding 10 g peptone, 5 g yeast extract, and 5 g NaCl, then sterilized in an autoclave at 15 psi and 121 °C for 15 min. For the LB solid medium, 15 g of bacteriological agar were added to 1 L of distilled water. *C. violaceum* CV026 was always grown in the presence of 30 μg/mL kanamycin. From the cryovials containing *C. violaceum* CV026, a roast was taken and crosswise streaked in a box with LB agar and 30 μg/mL kanamycin, which was incubated at 29 °C for 24 h. A roast was then taken from an isolated colony and inoculated in 5 mL LB medium with 30 μg/mL kanamycin for CV026, which was incubated at 29 °C and 200 rpm for 15 h. Finally, the boxes were stored in refrigeration. For each experiment, a cryo-stock was unthawed and cultured in 60 mL LB medium with 30 μg/mL kanamycin until reaching an optical density of 0.1 to 600 nm.

#### 3.2.5. Evaluation of Compounds as *Quorum Sensing* Inhibitors in *Chromobacterium violaceum* CV026

*C. violaceum* CV026 was cultured in 60 mL LB medium with 30 μg/mL kanamycin until reaching an optical density of 0.1 to 600 nm. Subsequently, in 2 mL tubes, 980 μL of this culture, 80 μM of C6-AHL (800 nM final concentration), and 10 μL of the dilutions of the test compounds were added until reaching the final concentrations of 1 mM, 100 μM, 10 μM, 1 μM, 100 nM, 10 nM, 1 nM, and 100 pM. Then, the tubes were incubated at 29 °C and 700 rpm for 24 h. Upon completion of the incubation, cell density was determined by absorbance at 720 nm by using LB medium as the blank. Finally, the absorbance of violacein was measured. Next, 500 μL of the bacterial culture were placed in a 2 mL tube and 500 μL of acetone were added. The tubes were vortexed and centrifuged at 15,000 rpm for 4 min, followed by determination of the absorbance of violacein in the supernatants at 577 nm. The readings at 577 nm and 720 nm were employed to calculate the specific production of violacein (dividing the value at 577 nm by that at 720 nm). Experiments were performed in duplicates.

#### 3.2.6. Statistical Analysis

The data of biofilm formation influenced by compounds **9**, **10**, **11**, **12,** and **14** in *Aeromonas caviae* Sch3 are presented as percentages of the biofilm formation of the control, which received no inhibitor treatment and was considered as 100%. Significance was confirmed by the Student’s *t*-test in Microsoft Office with an accuracy of * *p* < 0.005 and ^#^ *p* < 0.05 at the marked bars. Experiments were performed with *n* = 6. Figure 1.

The data of production of violacein by *Chromobacterium violaceum* CV026 under the influence of the added compounds **9**, **10**, **11**, **12**, and **14** were normalized on the violacein production without compound addition and this was considered as 100%. Experiments were performed as duplicates (Figure 2).

Experiments were performed with *n* = 6, and statistical significance was evaluated with the Student’s *t*-test in Microsoft Office.

## 4. Conclusions

Given the urgent need for new therapies to overcome the current bacterial antibiotic resistance problem, our study contributes to the research effort by providing a compound set and a valuable scaffold new for anti-QS in *Aeromonas caviae*. The introduction of alkyl-quinoxalin-2(1*H*)-one was shown to be successful in the reduction of biofilm formation. Within the hetero cyclic-integrated *N*-acyl structural solution, increased *N*-acyl chain length showed inhibition according to the current literature [7,9,21]. The substitution of the lactone moiety due to an aromatic feature has the potential for better metabolic stability due to the resistance against lactamases. The QS inhibition of these compounds was also confirmed in *Chromobacterium violaceum* by reduced violacein production. Based on our compound set, quinoxalin-2(1*H*)-one as anti-QS compounds could be further developed in the search for novel anti-infection treatments, whose importance is rapidly increasing, as anti-bacterial approaches keep losing effectiveness due to growing bacterial resistance. Future steps in the drug developing process would include the synthesis of more quinoxalin-2(1*H*)-one analogs to intensify the structure activity relationship study in *Aeromonas caviae*. The goal of this future work would be to improve the compound properties in terms of activity, water solubility, toxicity, selectivity, and bioavailability.

## Figures and Tables

**Table 1:** Inhibition of biofilm formation by anti-quorum sensing compounds. * *p* < 0.005, ^#^ *p* < 0.05.

	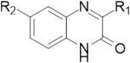		**% Reduction of Biofilm Formation**		
**ID**	**R_1_**	**R_2_**	100 µM	10 µM	1 µM
**9**	Ethyl	-	6	4	5
**10**	Butyl	-	3	4	3
**11**	Hexyl	-	25 *	11 ^#^	6
**12**	Hexyl	COOH	21 *	12 ^#^	1
**13**	Heptyl	-	-	-	-
**14**	Heptyl	COOH	59 *	24 *	19 *

## Supplementary Materials

^1^H NMR-, ^13^C NMR-, HSQC-, IR-, HRMS-spectra; control measurement for DMSO effect on biofilm formation.
