# Supplementary material for: Design, Synthesis, and Evaluation of Alkyl-Quinoxalin-2(1H)-One Derivatives as Anti-Quorum Sensing Molecules, Inhibiting Biofilm Formation in Aeromonas caviae Sch3
Source: Molecules. 2018 Nov 24;23(12):3075. doi: 10.3390/molecules23123075 (PMC6321446; doi:10.3390/molecules23123075)
Supplement: Supplementary file 1 [file molecules-23-03075-s001.pdf]

# Supplementary Material

## Selected spectra

### $^1H$ NMR

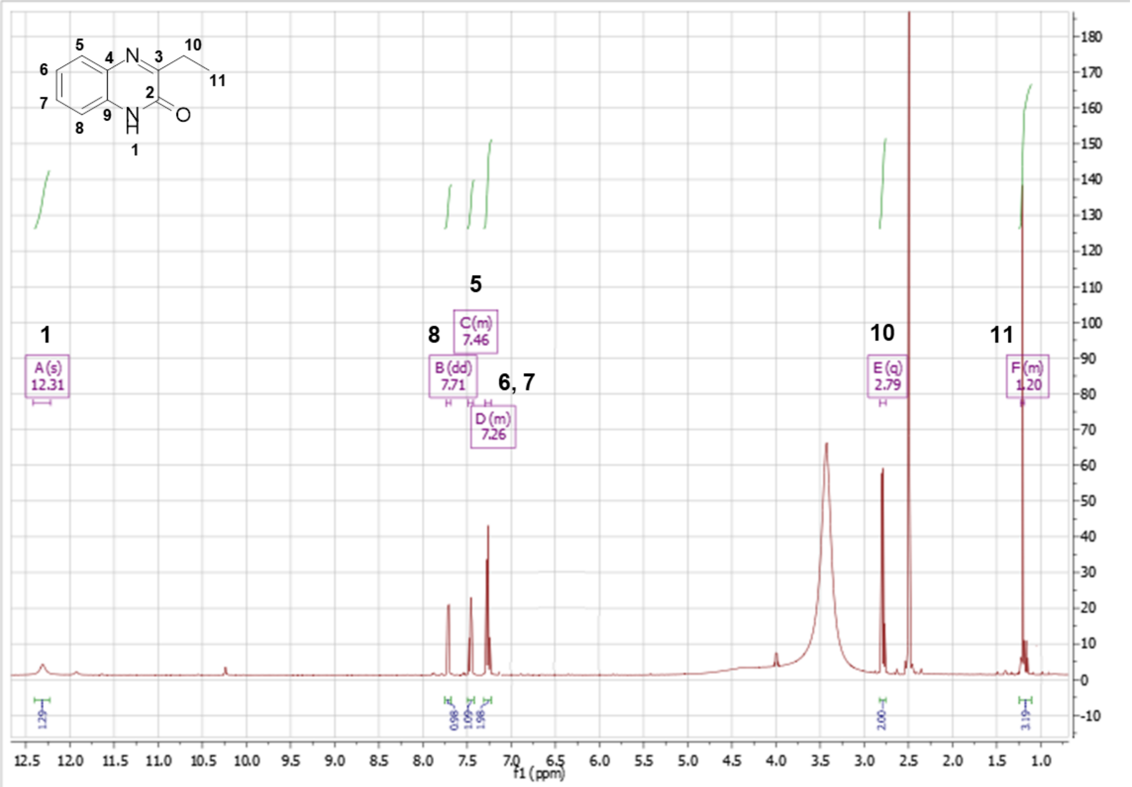


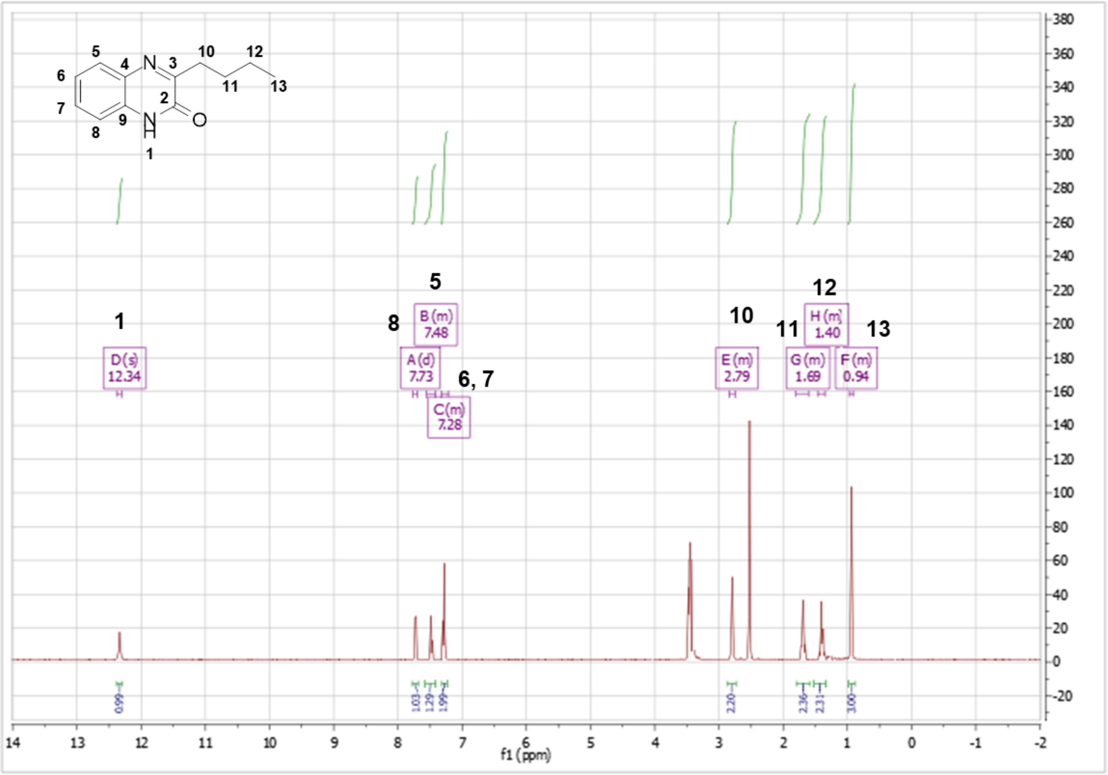

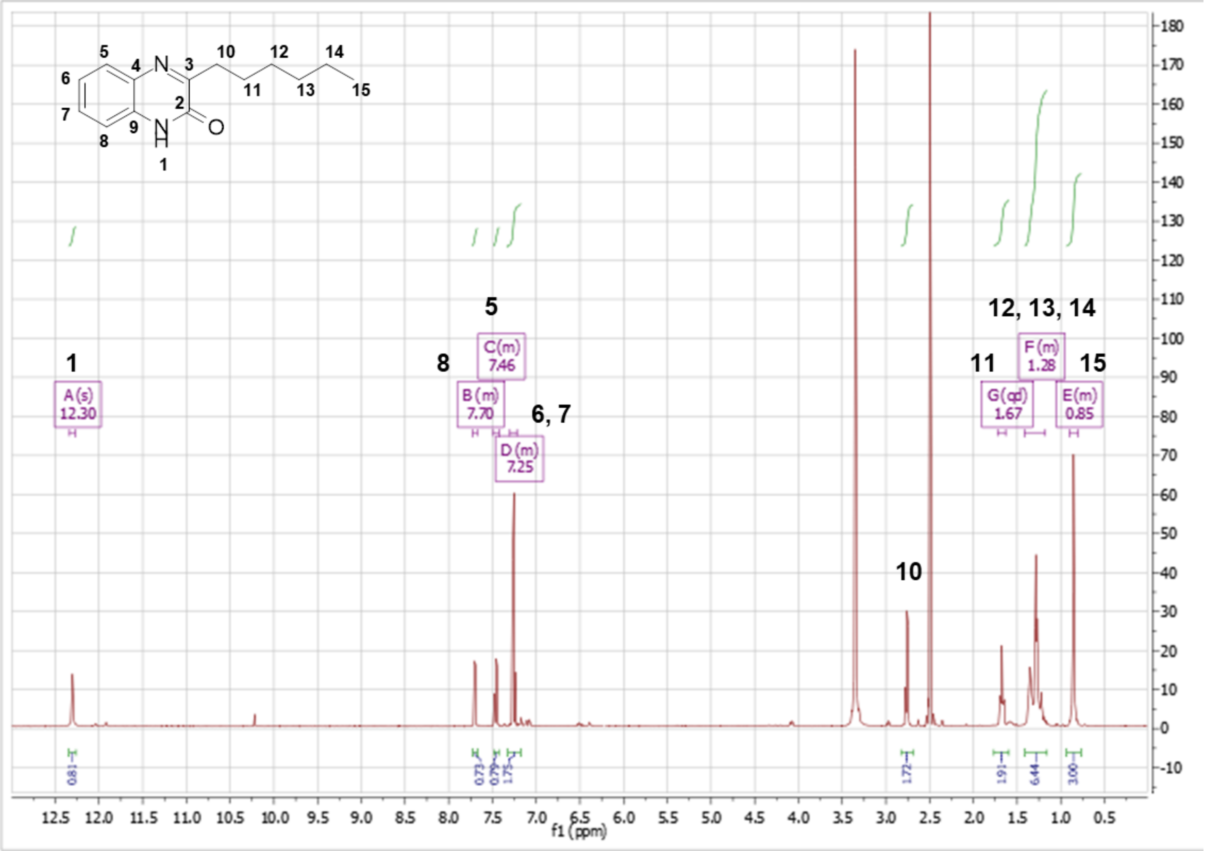
1
A (s)
12.30
8
B (m)
7.70
5
C (m)
7.46
6, 7
D (m)
7.25
10
11
G (qd)
1.67
12, 13, 14
F (m)
1.28
15
E (m)
0.85
0.81
0.73
0.79
1.75
1.72
1.91
6.44
3.00
f1 (ppm)

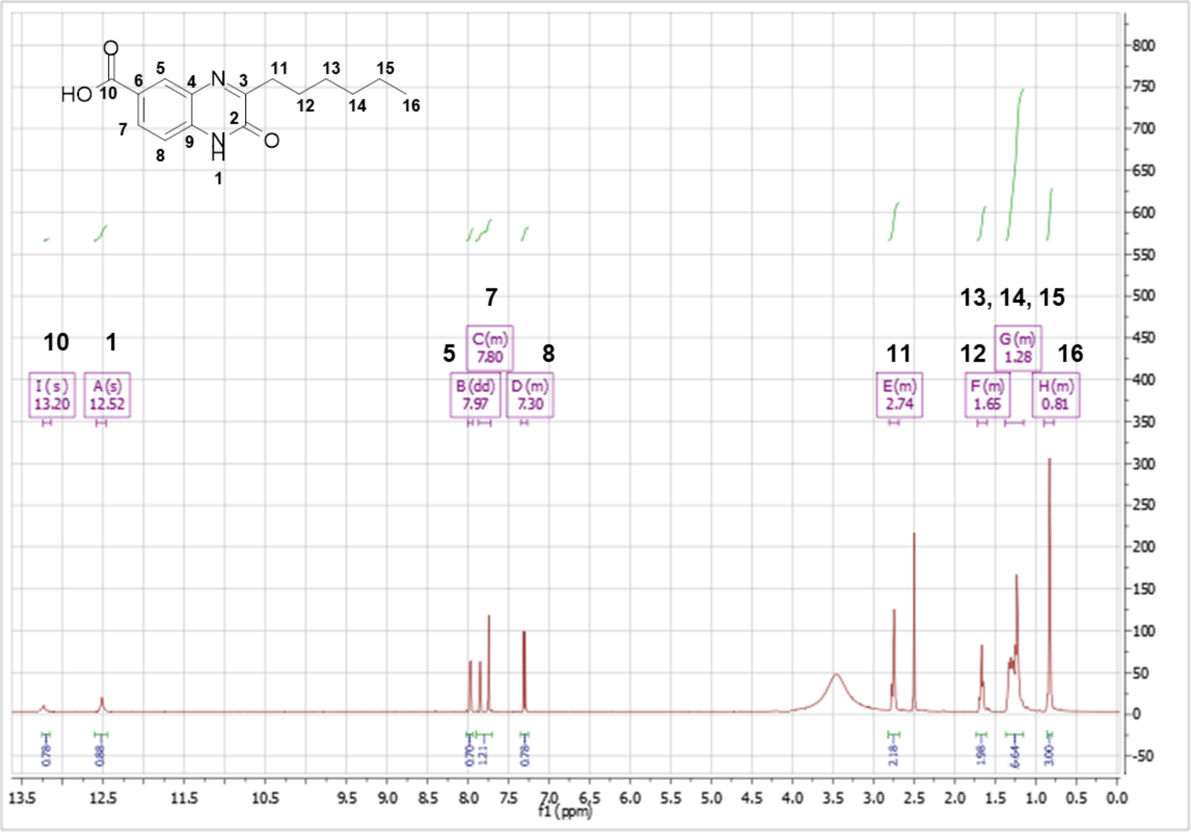
HO
10
1
I (s)
13.20
A (s)
12.52
5
B (dd)
7.97
7
C (m)
7.80
8
D (m)
7.30
11
E (m)
2.74
12
F (m)
1.65
13, 14, 15
G (m)
1.28
16
H (m)
0.81
0.78
0.88
0.70
1.21
0.78
2.18
1.98
6.64
3.00
f1 (ppm)

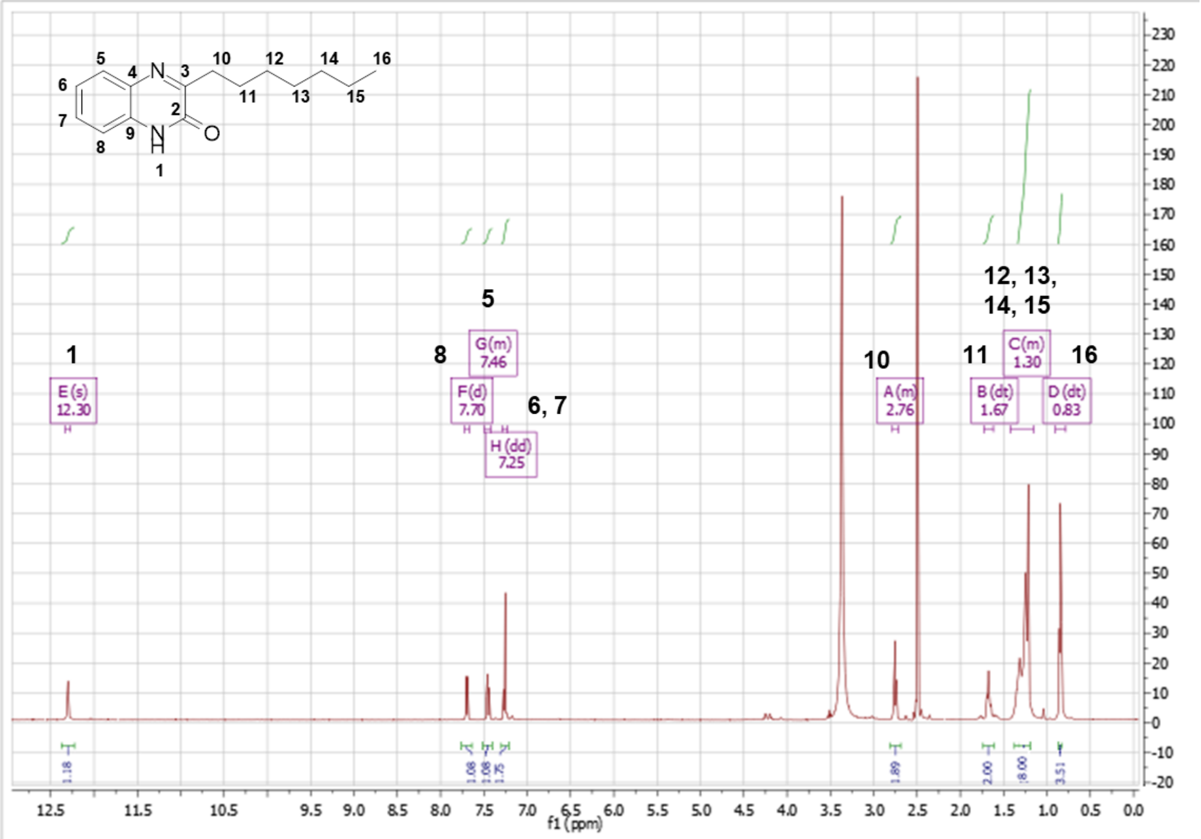

1
E (s) 12.30
8
F (d) 7.70
5
G (m) 7.46
6, 7
H (dd) 7.25
10
A (m) 2.76
11
B (dt) 1.67
12, 13, 14, 15
C (m) 1.30
16
D (dt) 0.83
1.18
1.08
1.08
1.75
1.89
2.00
8.00
3.51
f1 (ppm)


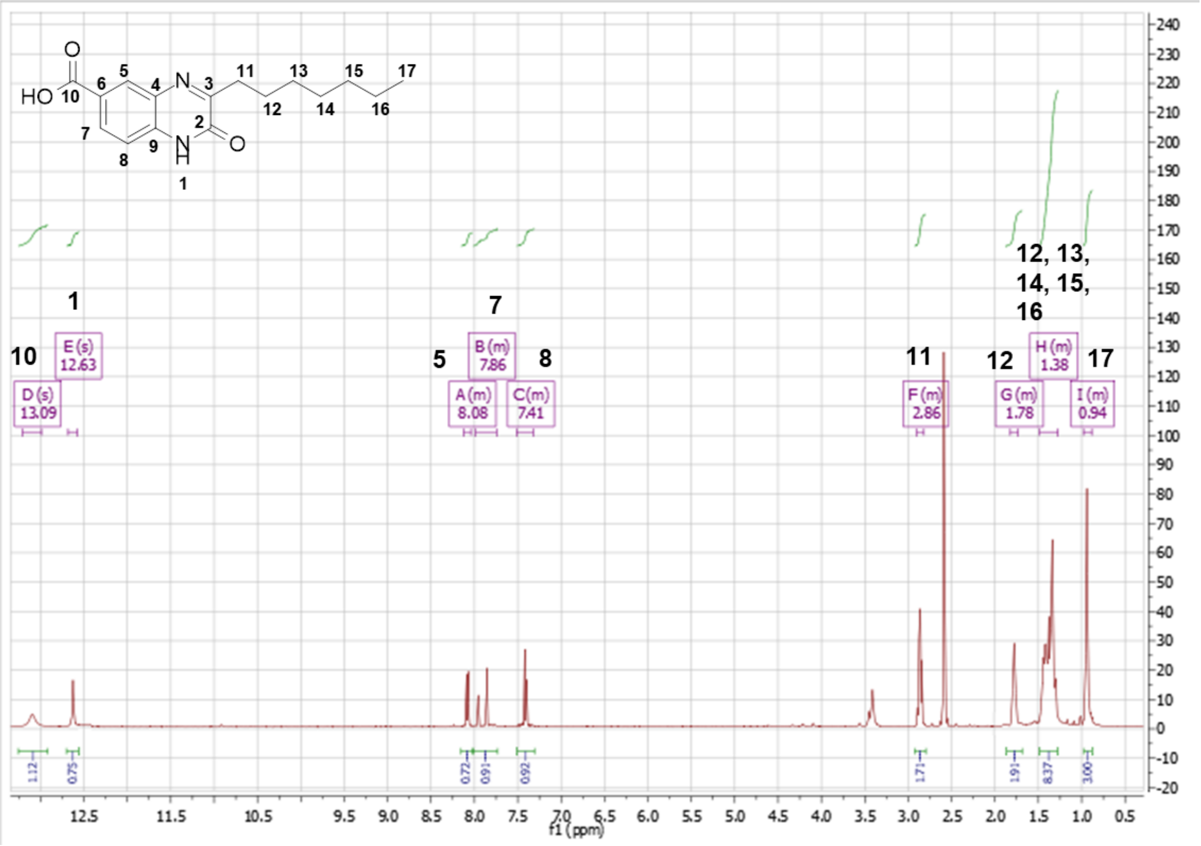

HO
10
D (s) 13.09
1
E (s) 12.63
5
A (m) 8.08
7
B (m) 7.86
8
C (m) 7.41
11
F (m) 2.86
12
G (m) 1.78
12, 13, 14, 15, 16
H (m) 1.38
17
I (m) 0.94
1.12
0.75
0.72
0.91
0.92
1.71
1.91
8.37
3.00
f1 (ppm)

$^{13}$C NMR spectras

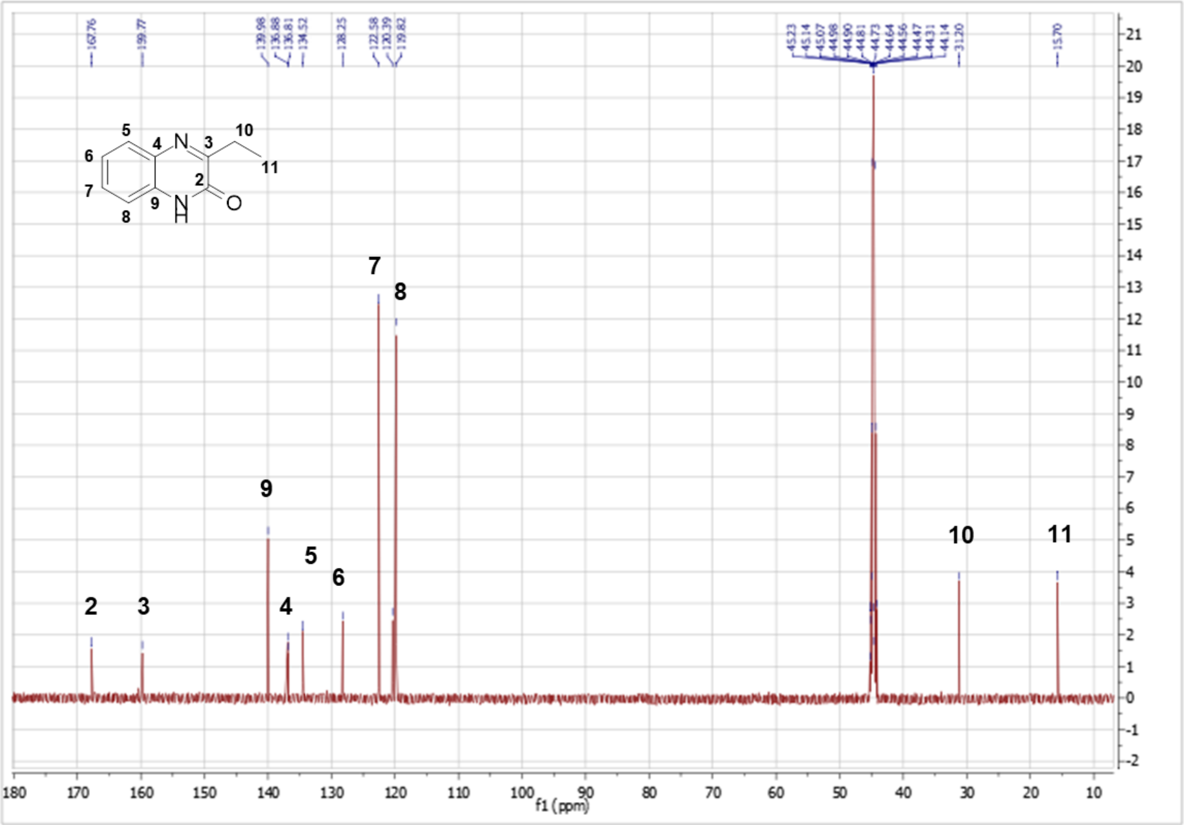


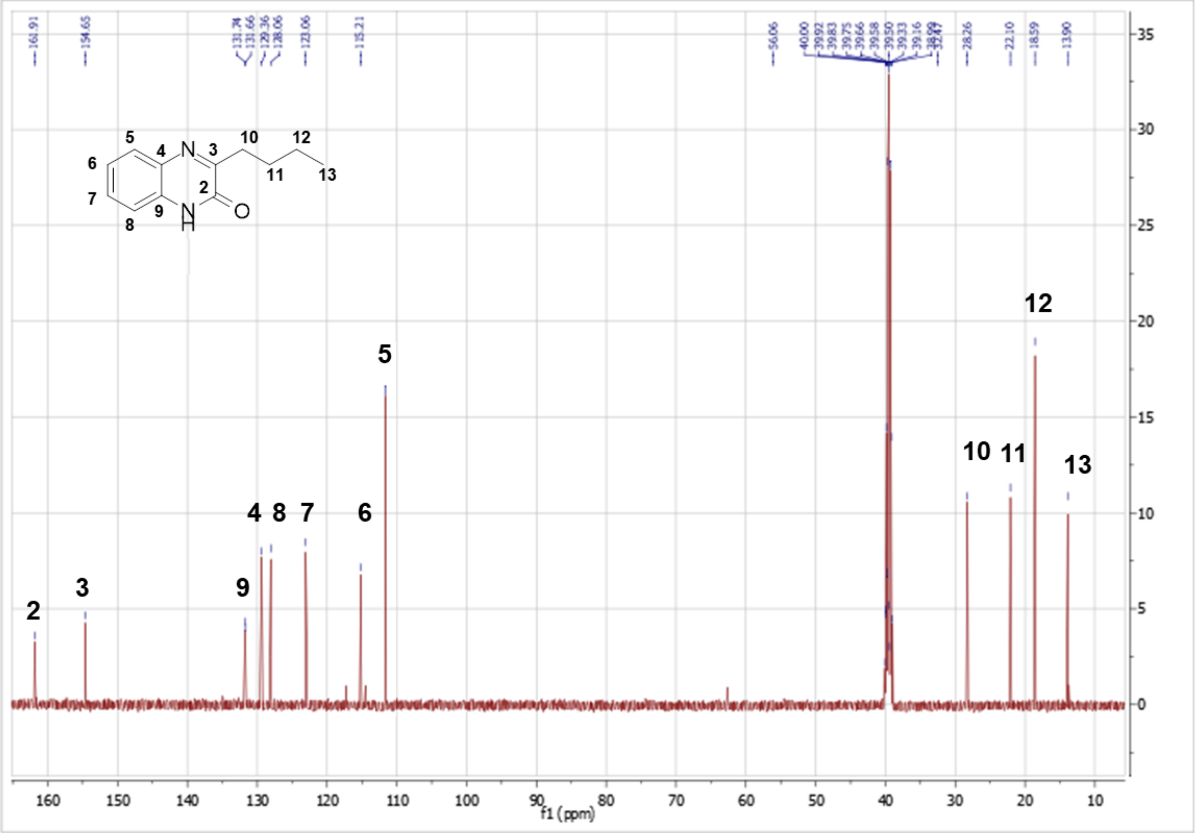

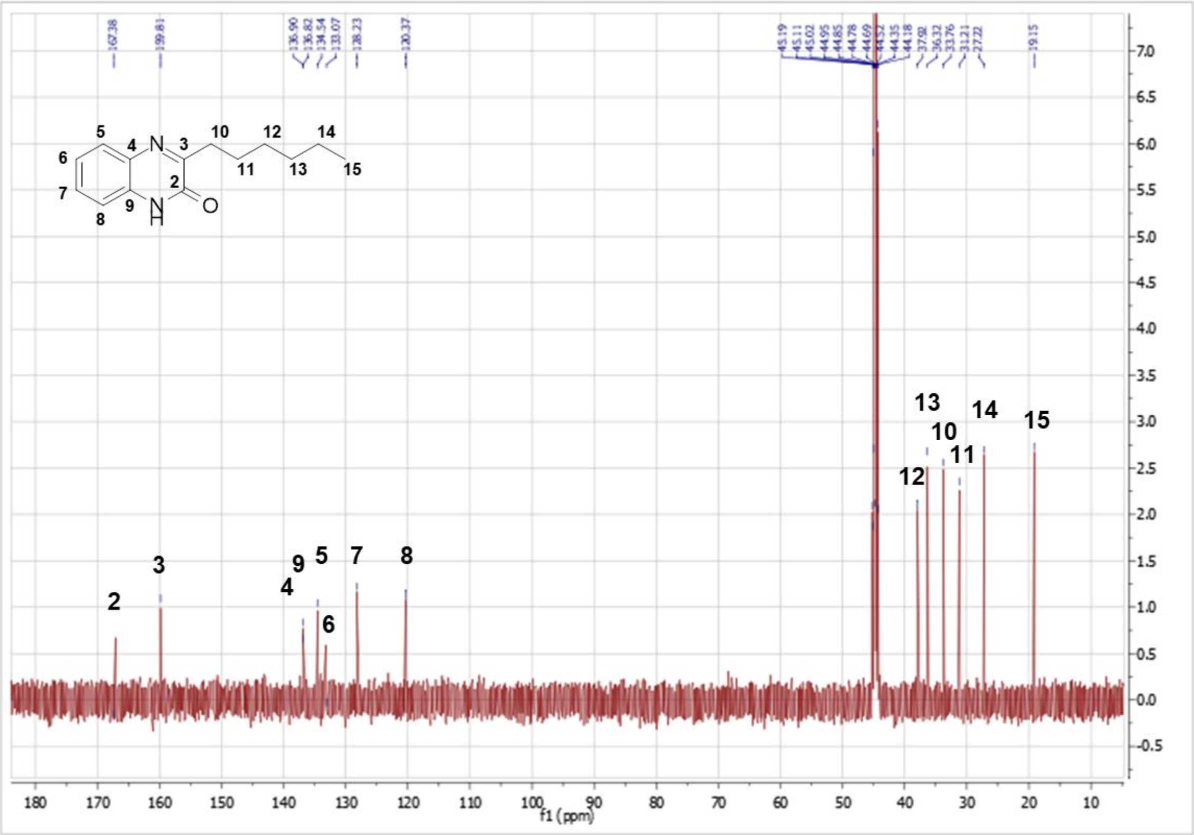
167.38
159.81
136.90
136.82
134.54
133.07
128.23
120.37
45.19
45.11
45.02
44.95
44.85
44.78
44.69
44.52
44.35
44.18
37.92
36.32
33.76
31.21
27.22
19.15
f1 (ppm)

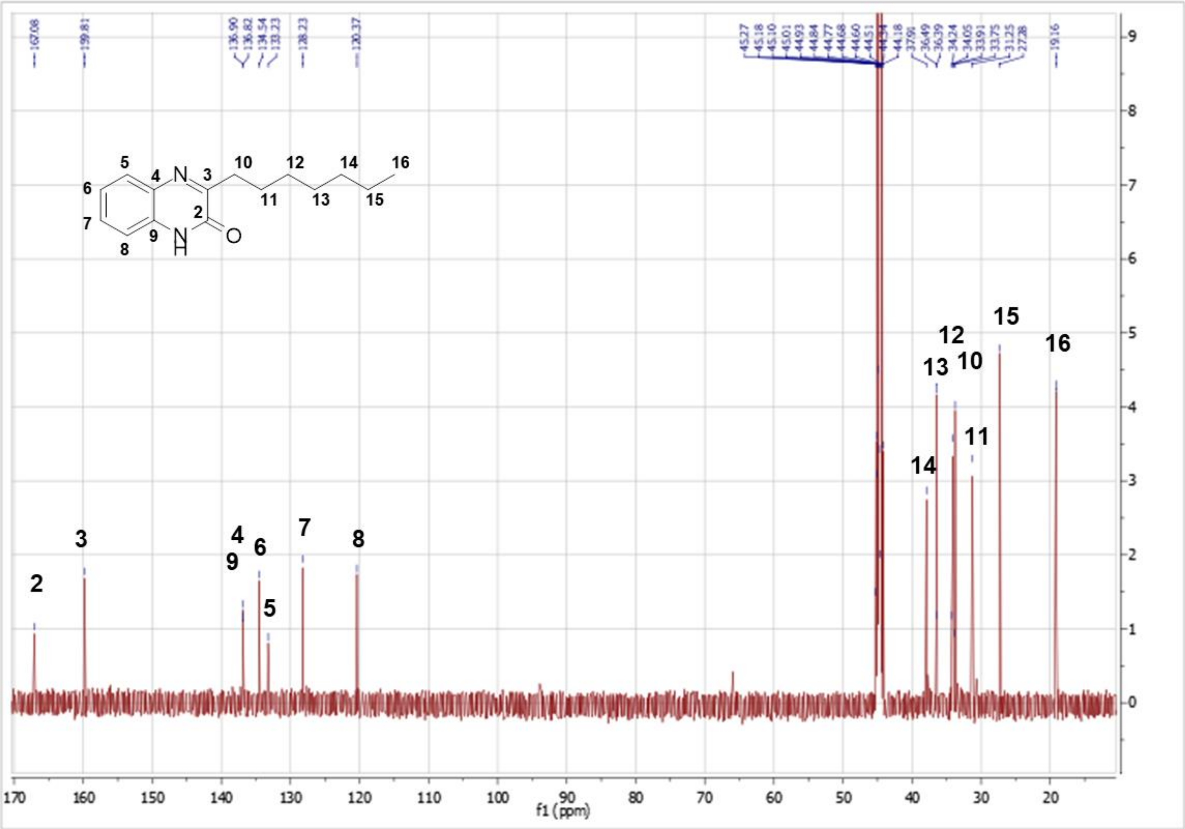
167.08
159.81
136.90
136.82
134.54
133.23
128.23
120.37
45.27
45.18
45.10
45.01
44.93
44.84
44.77
44.68
44.60
44.51
44.34
44.18
37.91
36.49
36.39
34.24
34.05
33.91
33.75
31.25
27.28
19.16
f1 (ppm)

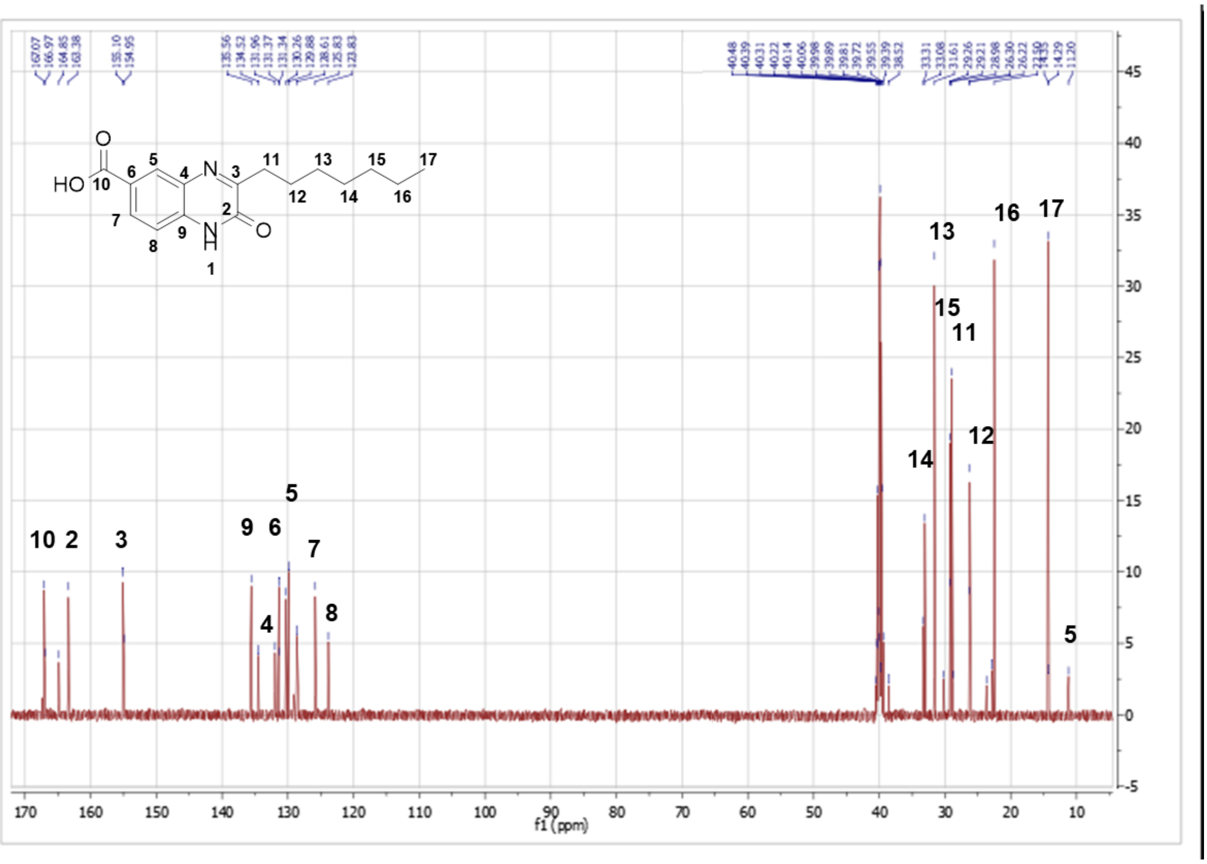


HSQC spectras

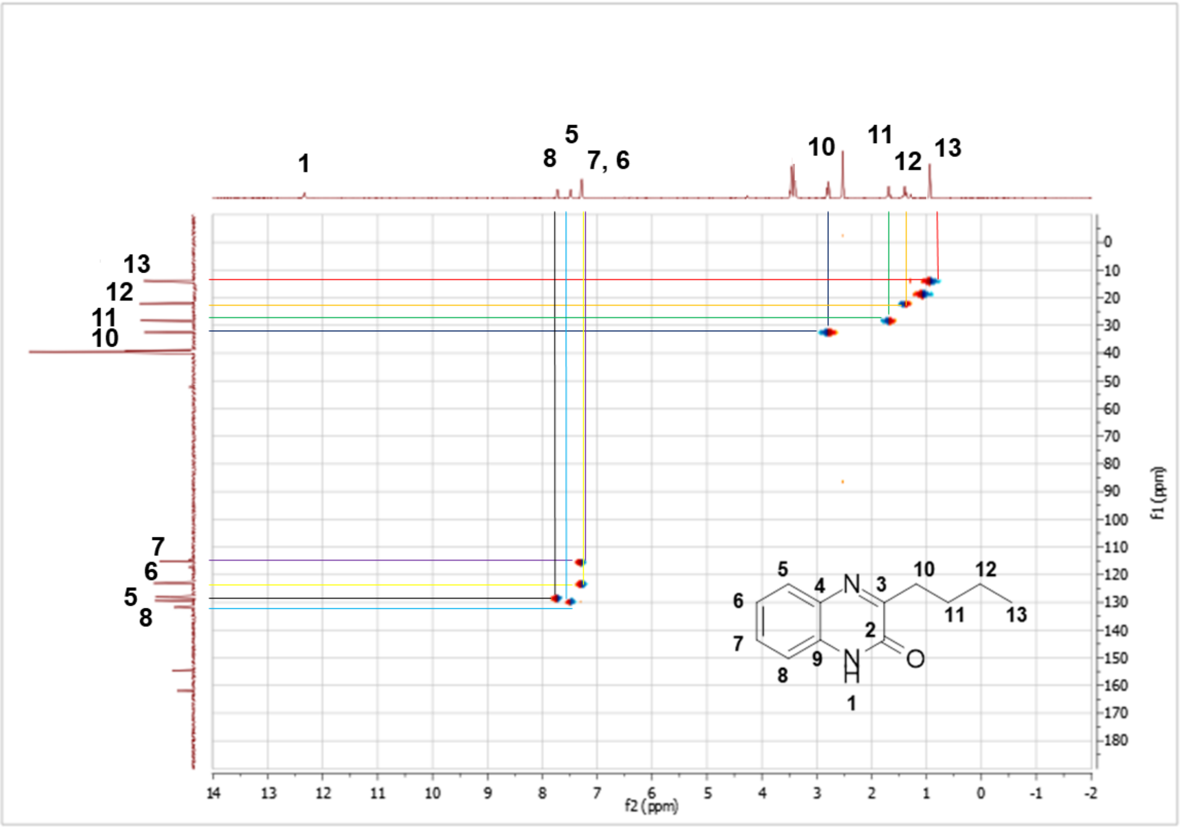

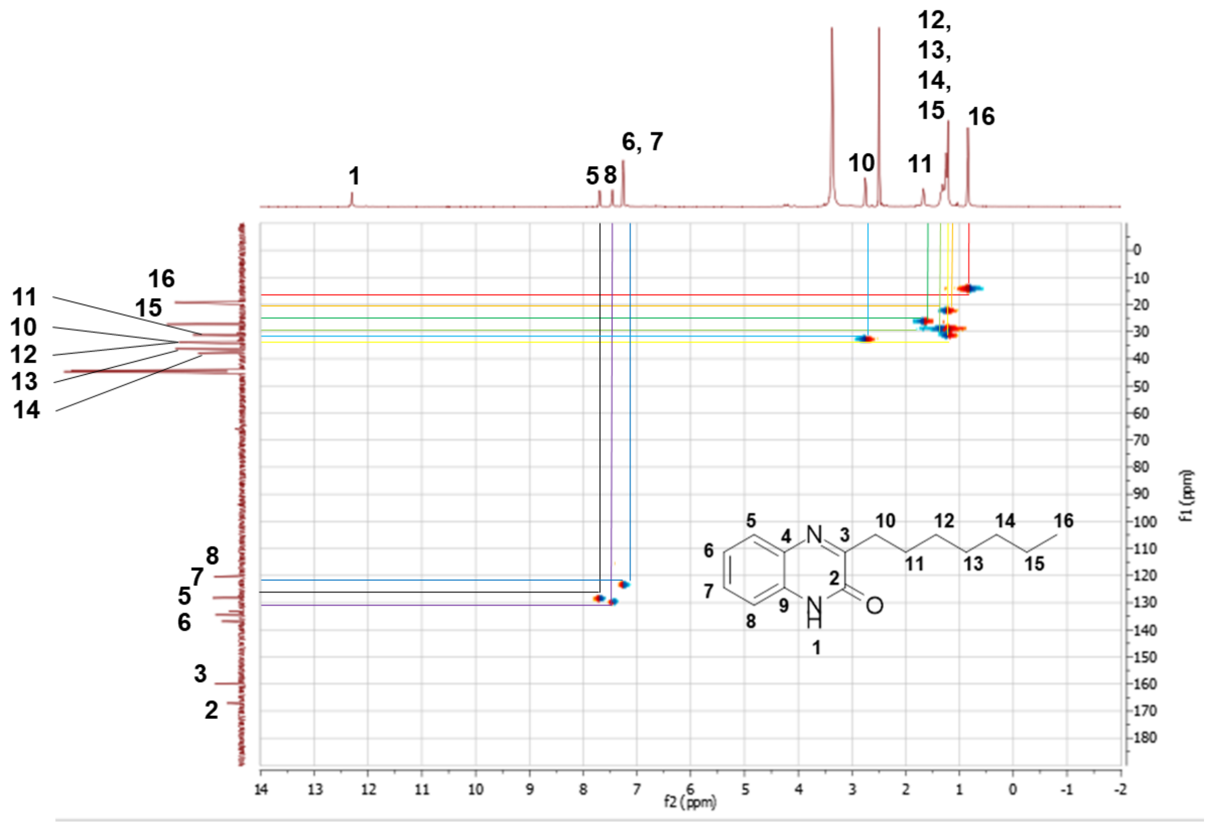

12,
13,
14,
15
16
6, 7
1
5 8
10
11
16
15
11
10
12
13
14
8
7
5
6
3
2
f2 (ppm)
f1 (ppm)


IR spectras

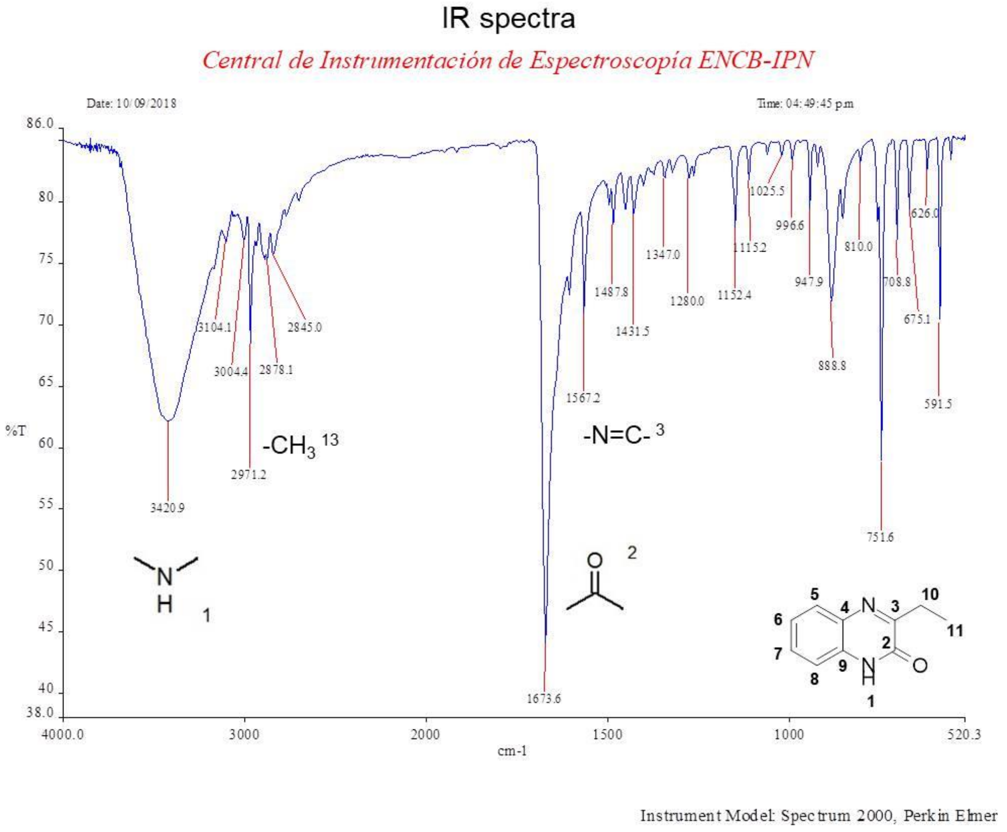

IR spectra
Central de Instrumentación de Espectroscopía ENCB-IPN
Date: 10/09/2018
Time: 04:49:45 p.m
%T
cm-1
3420.9
3104.1
3004.4
2971.2
2878.1
2845.0
1673.6
1567.2
1487.8
1431.5
1347.0
1280.0
1152.4
1115.2
1025.5
996.6
947.9
888.8
810.0
751.6
708.8
675.1
626.0
591.5
-CH3 13
-N=C- 3
Instrument Model: Spectrum 2000, Perkin Elmer

## IR spectra

*Central de Instrumentación de Espectroscopía ENCB-IPN*

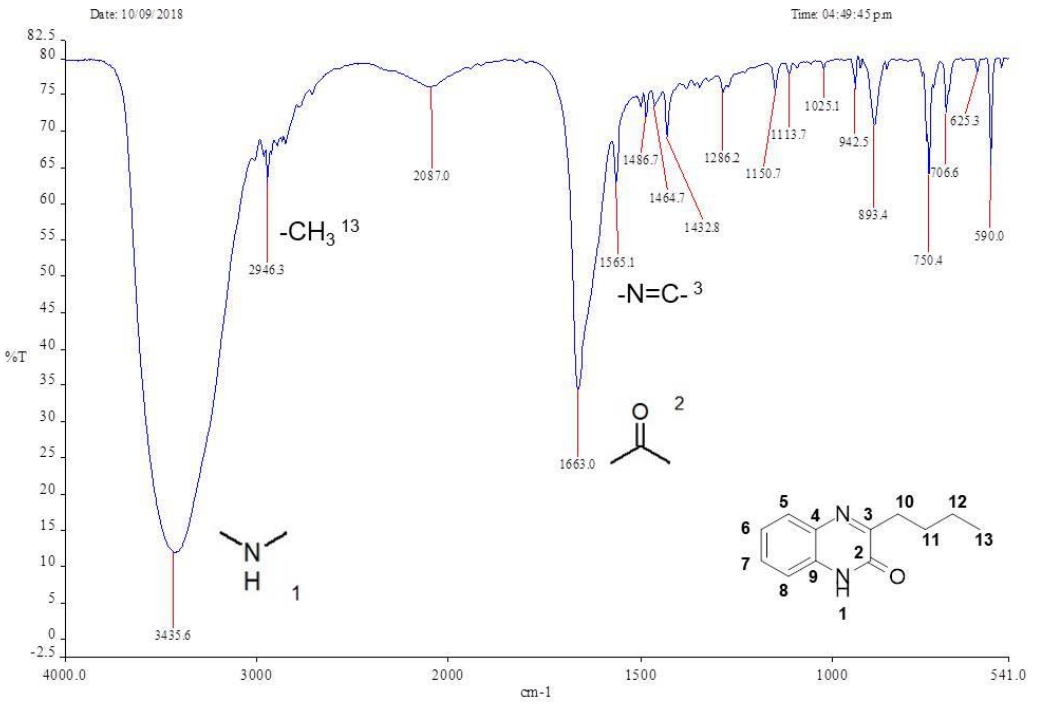


Instrument Model: Spectrum 2000, Perkin Elmer

## IR spectra

*Central de Instrumentación de Espectroscopía ENCB-IPN*

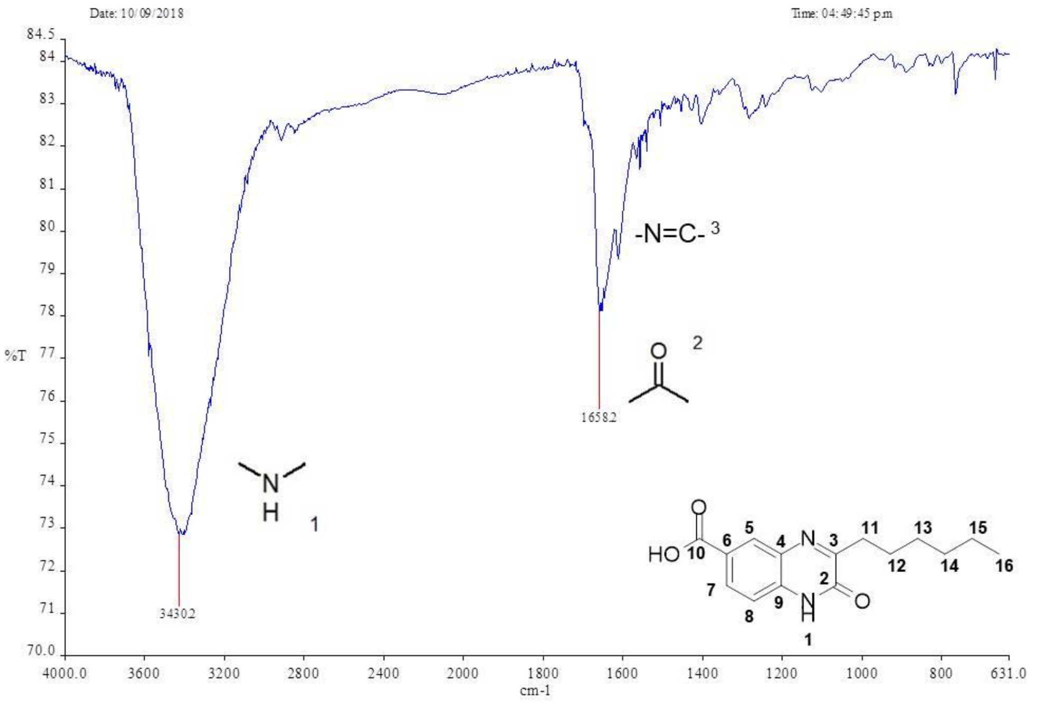


Instrument Model: Spectrum 2000, Perkin Elmer

## IR spectra

*Central de Instrumentación de Espectroscopía ENCB-IPN*

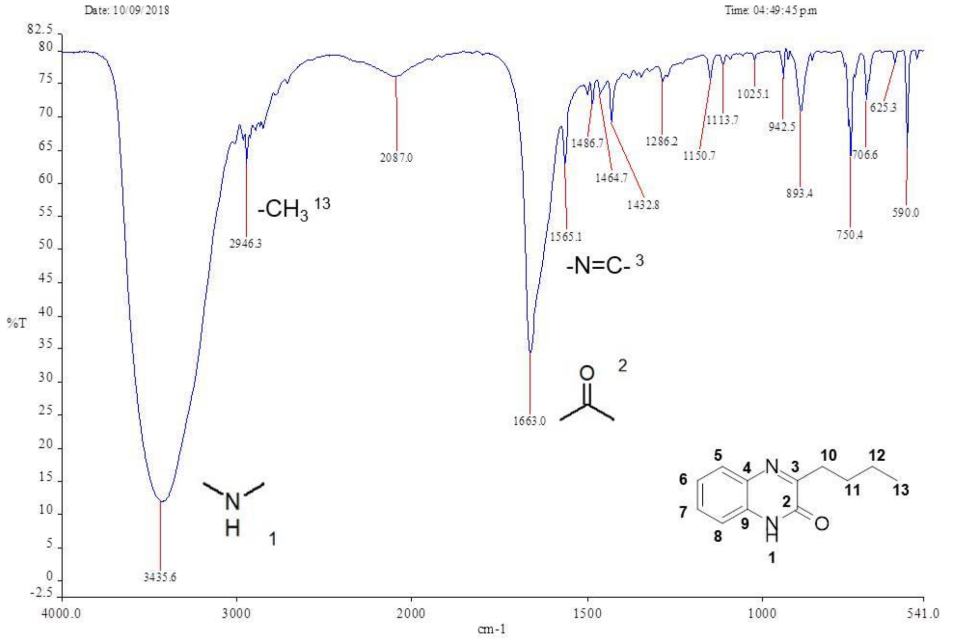


Instrument Model: Spectrum 2000, Perkin Elmer

## IR spectra

*Central de Instrumentación de Espectroscopía ENCB-IPN*

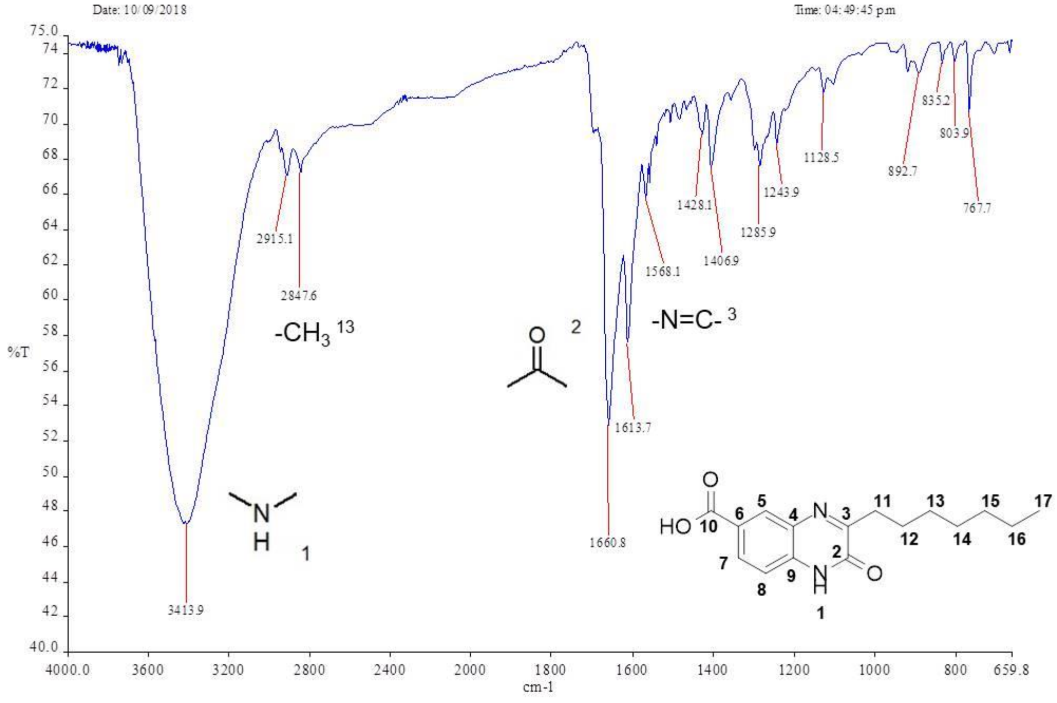


Instrument Model: Spectrum 2000, Perkin Elmer

HRMS spectras

# Display Report

**Analysis Info**

| | | | |
|---|---|---|---|
| Analysis Name | D:\Data\Alicia Reyes Arellano\092218_RBM1_.d | Acquisition Date | 9/22/2018 2:09:17 PM |
| Method | Tune_low_pos.m | Operator | Daniel Arrieta |
| Sample Name | 092218_RBM1_ | Instrument | micrOTOF-Q 228888.10392 |
| Comment | | | |

**Acquisition Parameter**

| | | | | | |
|---|---|---|---|---|---|
| Source Type | ESI | Ion Polarity | Positive | Set Nebulizer | 0.4 Bar |
| Focus | Active | Set Capillary | 4500 V | Set Dry Heater | 200 °C |
| Scan Begin | 50 m/z | Set End Plate Offset | -500 V | Set Dry Gas | 4.0 l/min |
| Scan End | 3000 m/z | Set Collision Cell RF | 250.0 Vpp | Set Divert Valve | Source |

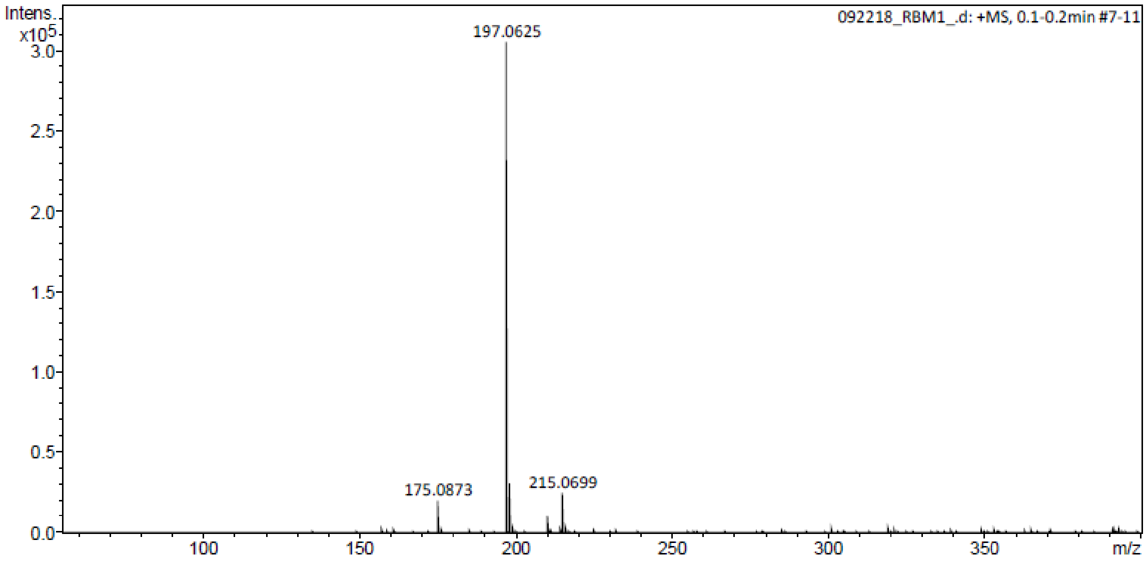


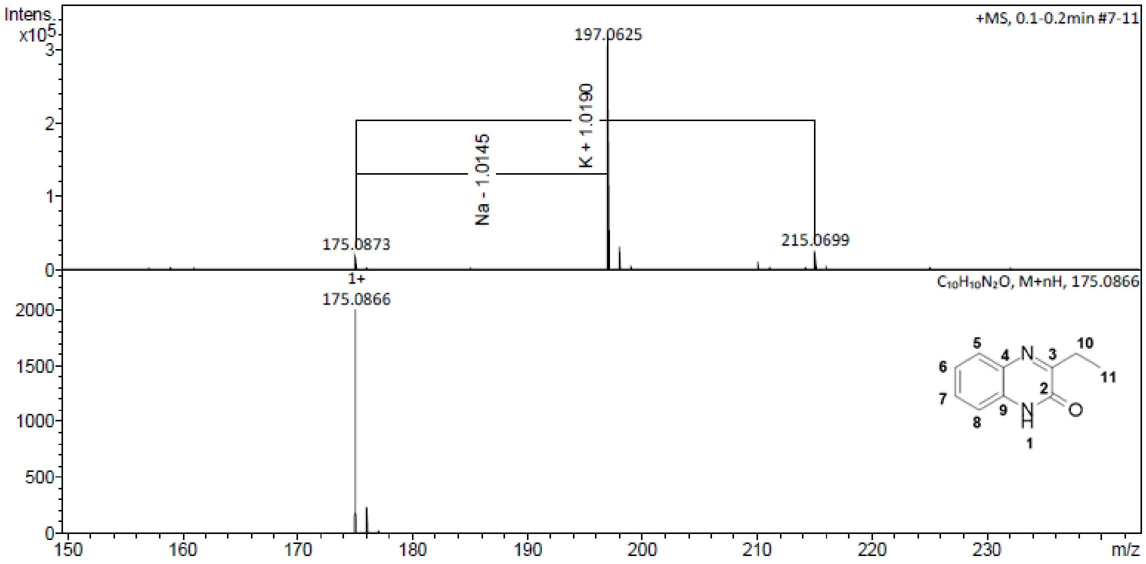

# Display Report

**Analysis Info**

| | | | |
|---|---|---|---|
| Analysis Name | D:\Data\Alicia Reyes Arellano\092218_RBM2_.d | Acquisition Date | 9/22/2018 2:18:06 PM |
| Method | Tune_low_pos.m | Operator | Daniel Arrieta |
| Sample Name | 092218_RBM2_ | Instrument | micrOTOF-Q 228888.10392 |
| Comment | | | |

**Acquisition Parameter**

| | | | | | |
|---|---|---|---|---|---|
| Source Type | ESI | Ion Polarity | Positive | Set Nebulizer | 0.4 Bar |
| Focus | Active | Set Capillary | 4500 V | Set Dry Heater | 200 °C |
| Scan Begin | 50 m/z | Set End Plate Offset | -500 V | Set Dry Gas | 4.0 l/min |
| Scan End | 3000 m/z | Set Collision Cell RF | 250.0 Vpp | Set Divert Valve | Source |

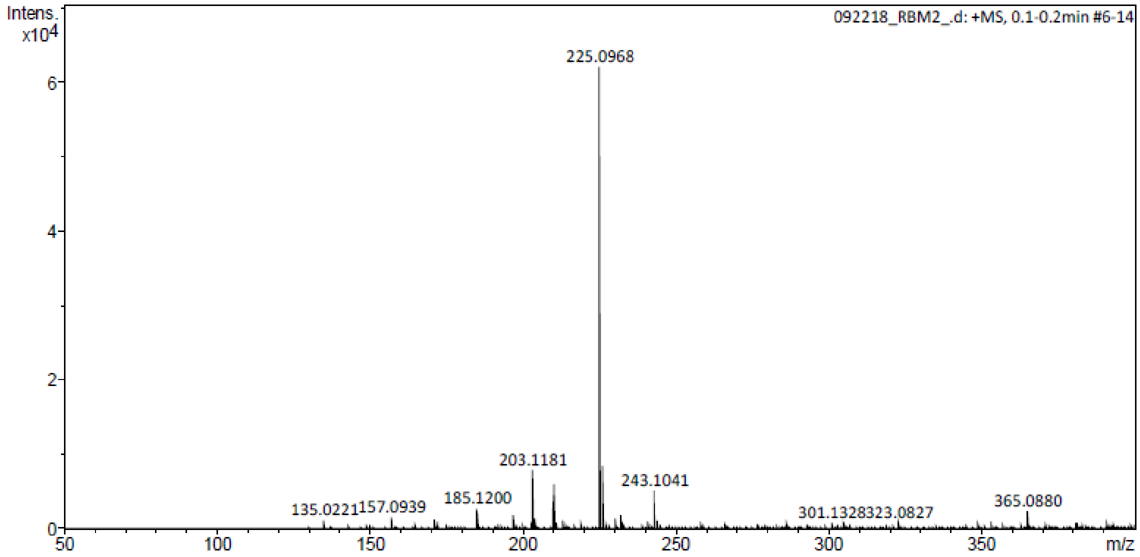


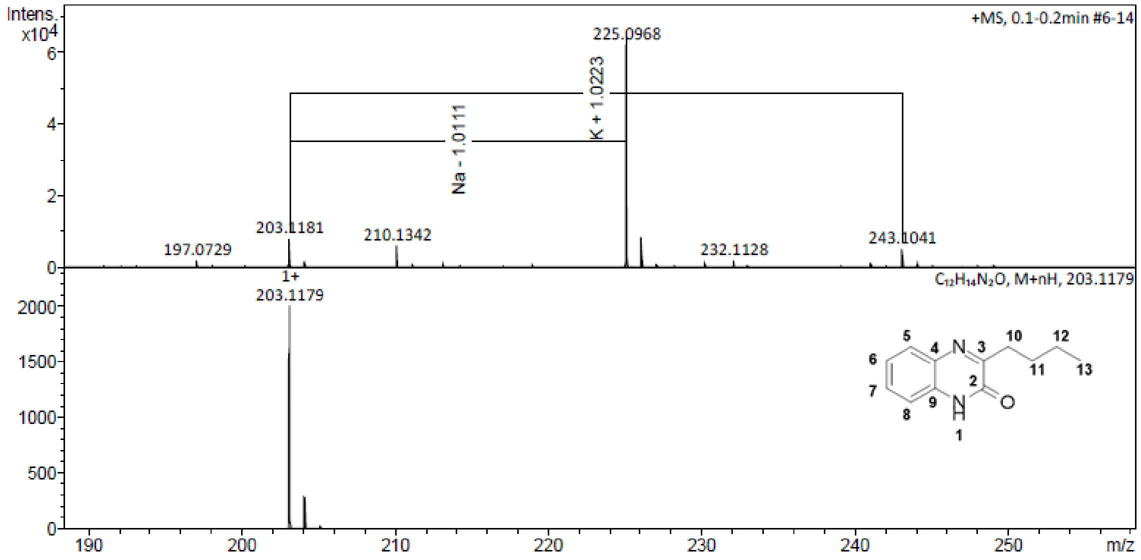

# Display Report

**Analysis Info**

| | | | |
|---|---|---|---|
| Analysis Name | D:\Data\Alicia Reyes Arellano\092218_RBM3.d | Acquisition Date | 9/22/2018 12:04:10 PM |
| Method | Tune low 2.m | Operator | Daniel Arrieta |
| Sample Name | 092218_RBM3 | Instrument | micrOTOF-Q 228888.10392 |
| Comment | | | |

**Acquisition Parameter**

| | | | | | |
|---|---|---|---|---|---|
| Source Type | ESI | Ion Polarity | Positive | Set Nebulizer | 0.4 Bar |
| Focus | Active | Set Capillary | 4500 V | Set Dry Heater | 180 °C |
| Scan Begin | 50 m/z | Set End Plate Offset | -500 V | Set Dry Gas | 4.0 l/min |
| Scan End | 3000 m/z | Set Collision Cell RF | 500.0 Vpp | Set Divert Valve | Source |

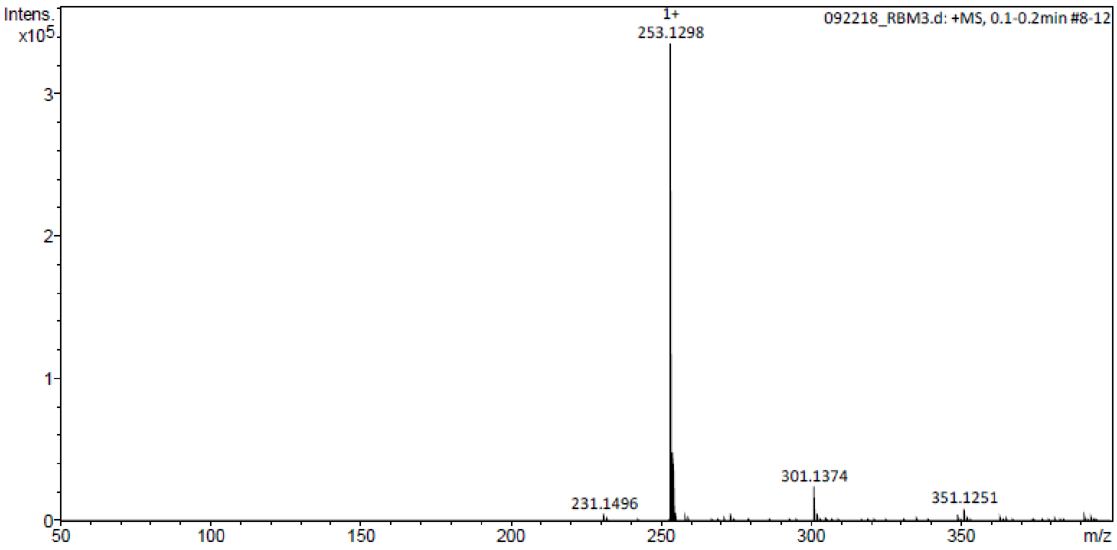


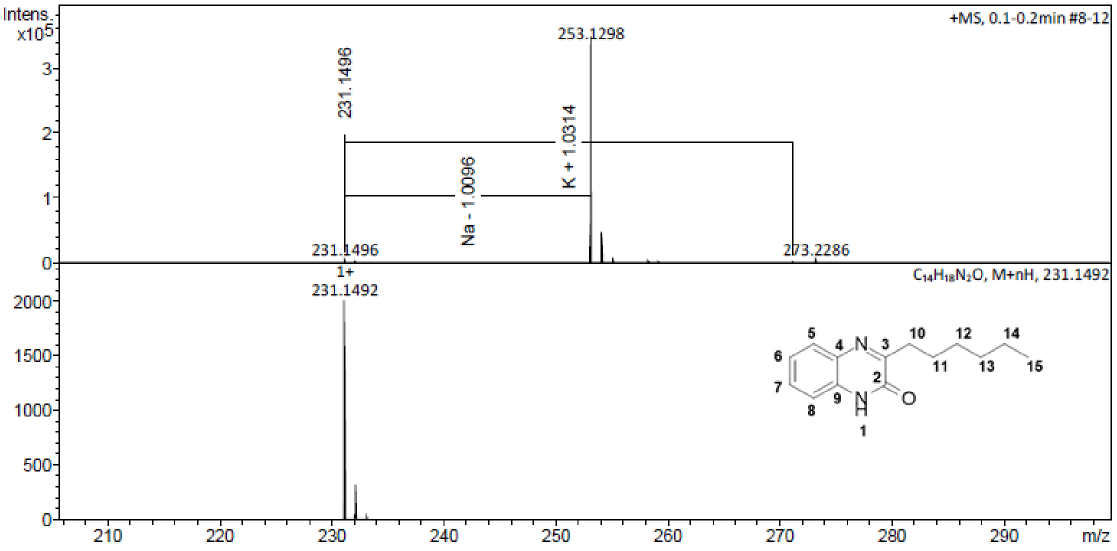

# Display Report

**Analysis Info**

| | | | |
|---|---|---|---|
| Analysis Name | D:\Data\Alicia Reyes Arellano\092218_RBM5.d | Acquisition Date | 9/22/2018 12:25:02 PM |
| Method | Tune low 2.m | Operator | Daniel Arrieta |
| Sample Name | 092218_RBM5 | Instrument | micrOTOF-Q 228888.10392 |
| Comment | | | |

**Acquisition Parameter**

| | | | | | |
|---|---|---|---|---|---|
| Source Type | ESI | Ion Polarity | Positive | Set Nebulizer | 0.4 Bar |
| Focus | Active | Set Capillary | 4500 V | Set Dry Heater | 180 °C |
| Scan Begin | 50 m/z | Set End Plate Offset | -500 V | Set Dry Gas | 4.0 l/min |
| Scan End | 3000 m/z | Set Collision Cell RF | 500.0 Vpp | Set Divert Valve | Source |

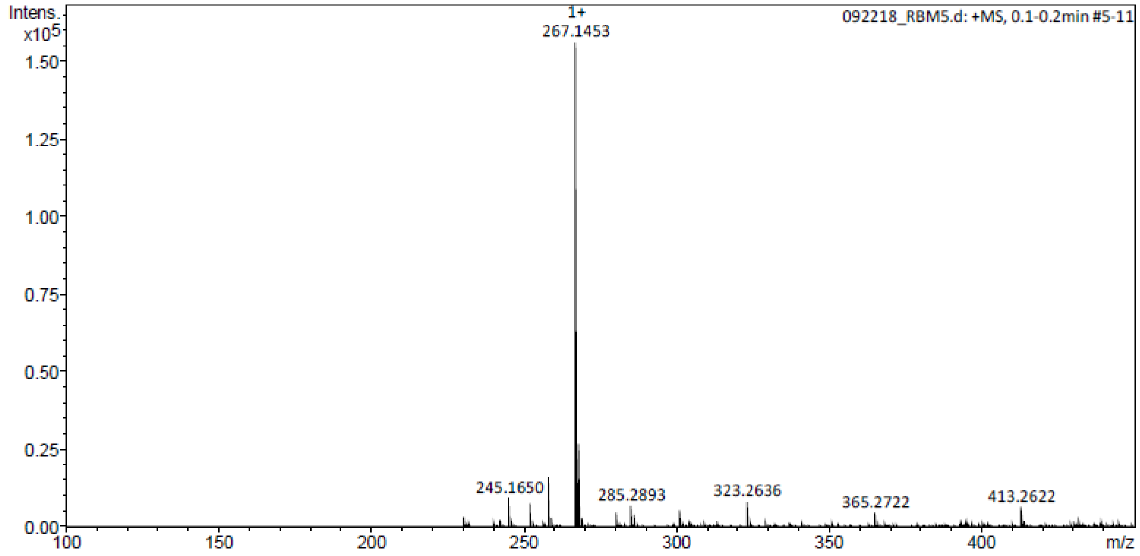


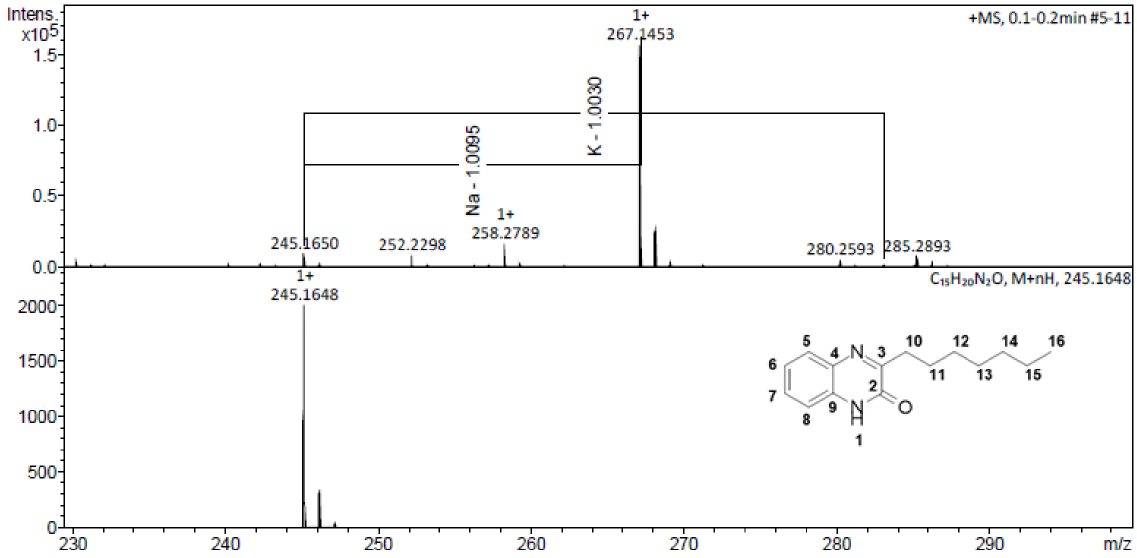

# Display Report

**Analysis Info**

| | | | |
|---|---|---|---|
| Analysis Name | D:\Data\Alicia Reyes Arellano\092218_RBM6.d | Acquisition Date | 9/22/2018 12:38:23 PM |
| Method | Tune low 2.m | Operator | Daniel Arrieta |
| Sample Name | 092218_RBM6 | Instrument | micrOTOF-Q 228888.10392 |
| Comment | | | |

**Acquisition Parameter**

| | | | | | |
|---|---|---|---|---|---|
| Source Type | ESI | Ion Polarity | Positive | Set Nebulizer | 0.4 Bar |
| Focus | Active | Set Capillary | 4500 V | Set Dry Heater | 200 °C |
| Scan Begin | 50 m/z | Set End Plate Offset | -500 V | Set Dry Gas | 4.0 l/min |
| Scan End | 3000 m/z | Set Collision Cell RF | 500.0 Vpp | Set Divert Valve | Source |

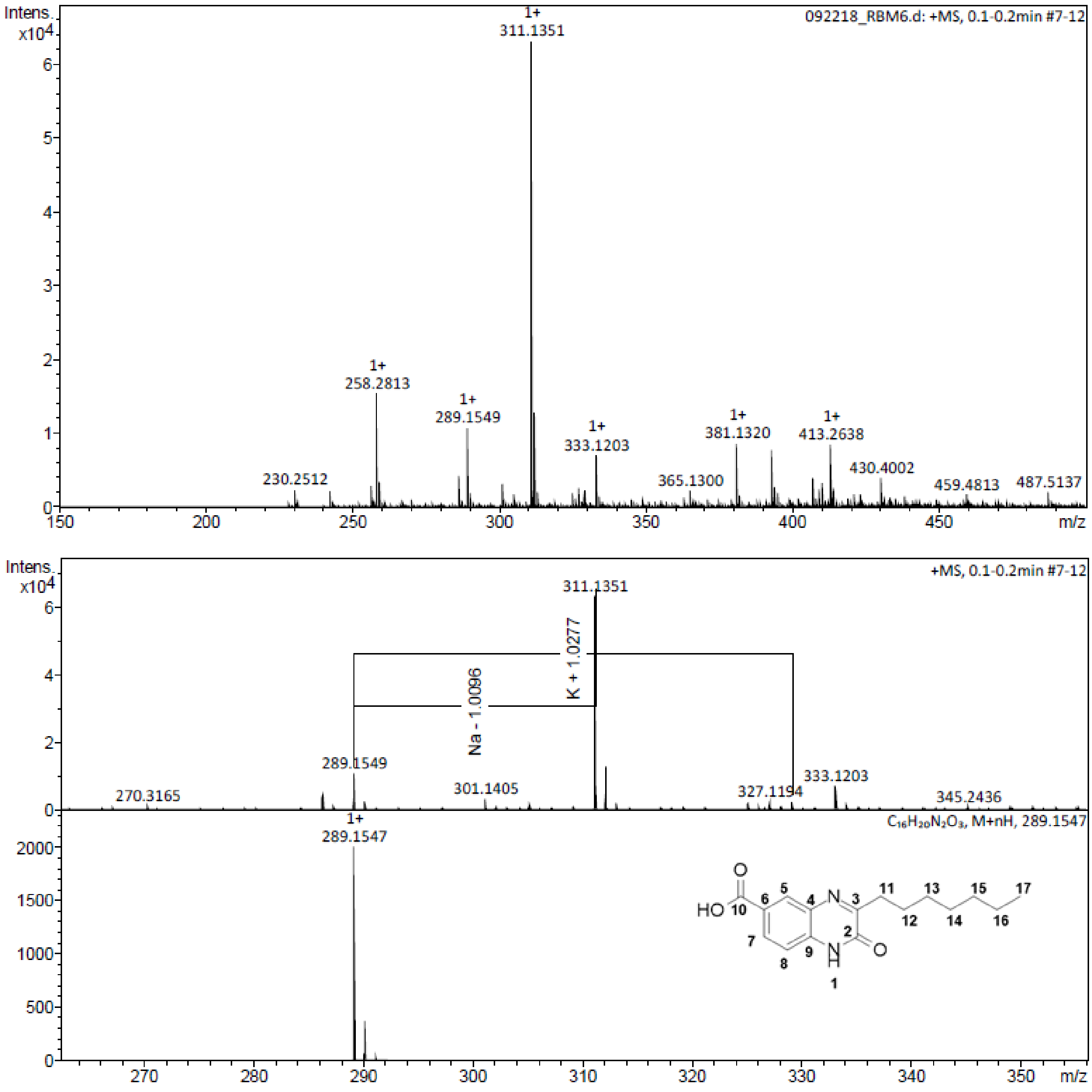

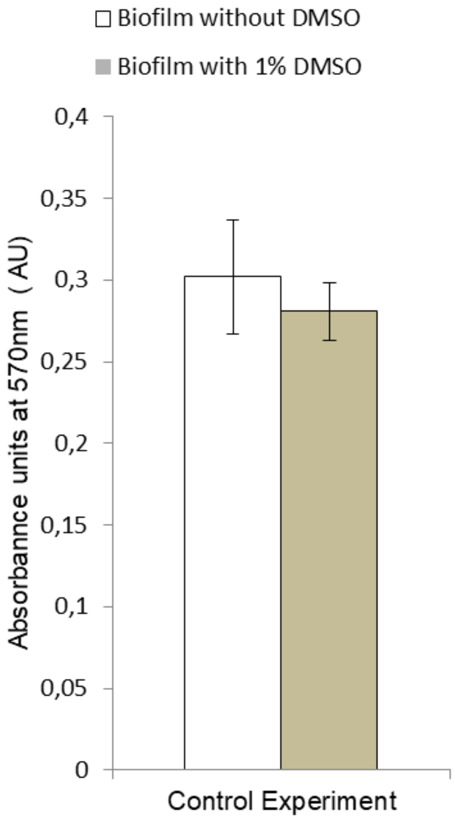


Control experiment confirming the insignificant effect of DMSO to biofilm formation in *Aeromonas cavia*.
